# Dual Charge Transfer Generated from Stable Mixed‐Valence Radical Crystals for Boosting Solar‐to‐Thermal Conversion

**DOI:** 10.1002/advs.202300980

**Published:** 2023-05-05

**Authors:** Jieqiong Xu, Jing Guo, Shengkai Li, Yanxia Yang, Weiming Lai, Phouphien Keoingthong, Shen Wang, Liang Zhang, Qian Dong, Zebing Zeng, Zhuo Chen

**Affiliations:** ^1^ Molecular Science and Biomedicine Laboratory (MBL) State Key Laboratory of Chemo/Biosensing and Chemometrics College of Chemistry and Chemical Engineering College of Biology Aptamer Engineering Center of Hunan Province Hunan University Changsha 410082 China; ^2^ State Key Laboratory of Chemo/Biosensing and Chemometrics College of Chemistry and Chemical Engineering College of Biology Hunan University Changsha Hunan 410082 China

**Keywords:** charge transfer, homoradical dimer, mixed‐valence, photothermal conversion, surfactants

## Abstract

Realizing dual charge transfer (CT) based on stable organic radicals in one system is a long‐sought goal, however, remains challenging. In this work, a stable mixed‐valence radical crystal is designed via a surfactant‐assisted method, namely TTF‐(TTF^+•^)_2_‐RC (where TTF = tetrathiafulvalene), containing dual CT interactions. The solubilization of surfactants enables successful co‐crystallization of mixed‐valence TTF molecules with different polarity in aqueous solutions. Short intermolecular distances between adjacent TTF moieties within TTF‐(TTF^+•^)_2_‐RC facilitate both inter‐valence CT (IVCT) between neutral TTF and TTF^+•^, and inter‐radical CT (IRCT) between two TTF^+•^ in radical *π*‐dimer, which are confirmed by single‐crystal X‐ray diffraction, solid‐state absorption, electron spin resonance measurements, and DFT calculations. Moreover, TTF‐(TTF^+•^)_2_‐RC reveals an open‐shell singlet diradical ground state with the antiferromagnetic coupling of 2J = −657 cm^−1^ and an unprecedented temperature‐dependent magnetic property, manifesting the main monoradical characters of IVCT at 113–203 K while the spin‐spin interactions in radical dimers of IRCT are predominant at 263–353 K. Notably, dual CT characters endow TTF‐(TTF^+•^)_2_‐RC with strong light absorption over the full solar spectrum and outstanding stability. As a result, TTF‐(TTF^+•^)_2_‐RC exhibits significantly enhanced photothermal property, an increase of 46.6 °C within 180 s upon one‐sun illumination.

## Graphical abstract

A stable mixed‐valence radical crystal (TTF‐(TTF^+•^)_2_‐RC) with dual charge transfer (CT) is synthesized via a surfactant‐assisted method, containing inter‐valence CT between TTF and TTF^+•^ and inter‐radical CT in TTF^+•^ dimer. Dual CT characters endow TTF‐(TTF^+•^)_2_‐RC with strong full‐spectrum light absorption that can effectively convert solar‐energy into heat, an increase of 46.6 °C within 180 s upon one‐sun illumination.

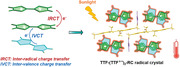

## 1 Introduction

Organic charge transfer (CT) materials, no matter with intermolecular or intramolecular CT transitions, are attracting increasing attention due to their fascinating application prospects in organic conductors,^[^ ^1^ ^]^ solid‐state luminescence,^[^ ^2^ ^]^ solar energy conversion,^[^ ^3^ ^]^ etc. Specially designed closed‐shell molecules are often acted as electron donors or acceptors to build high‐performance organic functional materials.^[^ ^4^ ^]^ Except from establishing strong electron delocalization between designed closed‐shell molecules, the direct use of stable open‐shell radicals to participate in CT is also a straightforward and effective way to achieve ground‐breaking improvements in performance.^[^ ^5^ ^]^ For instance, stable open‐shell donors, including dithiadiazolyl^[^ ^6^ ^]^ and verdazyl^[^ ^7^ ^]^ neutral *π*‐radicals are used for the construction of highly conductive CT salts due to their inherent half‐filled bands formed by *π*‐orbital overlap. On the other hand, open‐shell acceptors represented by tris‐2,4,6‐trichlorophenylmethyl (TTM) radicals^[^ ^8^ ^]^ are widely applied to organic deep‐red/near‐infrared emission by introducing electron‐donating substituent groups, because the CT state can improve the electron or hole transport abilities and thus enhance the quantum efficiency and stability.^[^ ^9^ ^]^ Recently, we have constructed a novel CT cocrystal by using persistent 2,2′‐azino‐bis‐(3‐ethylbenzothiazoline‐6‐sulfonic acid) cation radical (ABTS^+•^) with long‐wavelength absorption as electron acceptor and achieved high‐efficiency solar‐thermal conversion.^[^ ^10^ ^]^

Besides these CT forms between organic radicals and heterogeneous donors or acceptors, there are two other unique CT forms based on homogeneous radical molecules in material science. One is the inter‐valence charge transfer (IVCT)^[^ ^11^ ^]^ between neutral parent molecules and ion‐radicals. Strong electronic coupling between neighboring species could result in high electron delocalization within the dimers, trimers or higher‐order associations, which makes organic mixed‐valence salts with high mobility of the charge carriers in conducting solids.^[^ ^1^, ^12^ ^]^ Another is the inter‐radical charge transfer (IRCT)^[^ ^13^ ^]^ between two radicals in homoradical *π*‐dimers. The unconventional *π*‐stacking pattern composed of the multicentred *π*‐bonded dimers of conjugated organic radicals enables two unpaired electrons to undergo spin‐pairing and thus form diamagnetic (insulator state) radical dimers.^[^ ^14^ ^]^ Mixed‐valence dimers and radical dimers are, however, weakly associated species and usually exist in the solid at low temperature.^[^ ^15^ ^]^ Different strategies including encapsulation in a confined space,^[^ ^16^ ^]^ immobilized by metal–organic frameworks (MOF),^[^ ^17^ ^]^ and mechanically interlocked molecules (MIMs)^[^ ^18^ ^]^ have been employed to stabilize these radical derivatives. Nevertheless, few studies on how to achieve simultaneous IVCT and IRCT in one system are reported so far.

Owing to the superb electron‐donating capability of neutral tetrathiafulvalene (TTF) and its two stable and reversible oxidation states (i.e., cation radical TTF^+•^ and dication TTF^2+^), TTF and its analogs have attracted tremendous attention in organic solid‐state material applications.^[^ ^1^, ^19^ ^]^ The single‐electron oxidized form, TTF^+•^, can either undergo IVCT transition with neutral TTF to form valence dimer [TTF_2_]^+•^, or undergo IRCT transition with itself to form the homoradical *π*‐dimer [TTF^+•^]_2_, respectively.^[^ ^20^ ^]^ However, realizing the coexistence of IVCT and IRCT based on TTF^+•^ in one system is still a challenge. Cocrystal engineering composed by simple constituent units with a noncovalent assembly and simple synthetic procedure feature has emerged as an effective and versatile way to create organic functional materials.^[^ ^21^ ^]^ Benefitting from the effective CT and the diversity of components, organic cocrystals can be designed with flexible light absorption to meet different application requirements, which is difficult to achieve with traditional photothermal materials.^[^ ^22^ ^]^ Moreover, precise cocrystal architectures offer the opportunity to unveil the structure–property and charge transfer–property relationships.^[^ ^23^ ^]^

In this work, we design a stable mixed‐valence radical crystal, 2TTF‐2TTF^+•^‐2,2′‐azino‐bis‐(3‐ethylbenzothiazoline‐6‐sulfonic acid) (ABTS)‐2H_2_O, namely TTF‐(TTF^+•^)_2_‐RC, containing both IVCT and IRCT by using surfactant‐mediated cocrystallization method. The persistent ABTS^+•^ is utilized to oxidize TTF into TTF^+•^ through one‐electron oxidation, and the reduced state ABTS can also serve as counterions in crystals. With the solubilization of surfactants polyoxyethylene (100) stearyl ether (C_18_‐PEG), excess hydrophobic TTF are dispersed in water and favorable to cocrystallize quickly with two redox products. Single‐crystal X‐ray diffraction (SC‐XRD) analysis demonstrates that TTF‐(TTF^+•^)_2_‐RC contains a unique TTF dicationic trimer, consisting of one neutral TTF and one TTF^+•^ homoradical *π*‐dimer. In TTF‐(TTF^+•^)_2_‐RC, short intermolecular distances between neighboring TTF moiety within the trimer facilitate both IVCT between TTF and TTF^+•^, and IRCT between two TTF^+•^ in dimer, which are confirmed by a series of experiments and DFT calculations. Notably, dual CT characters endow TTF‐(TTF^+•^)_2_‐RC with appreciable full‐spectrum light absorption, and thus display remarkable photothermal conversion, an increase of 46.6 °C within 180 s under one‐sun illumination.

## 2 Results and Discussion

### 2.1 Surfactant‐Assisted Synthesis of the Radical Crystal TTF‐(TTF^+•^)_2_‐RC

**Figure 1:**
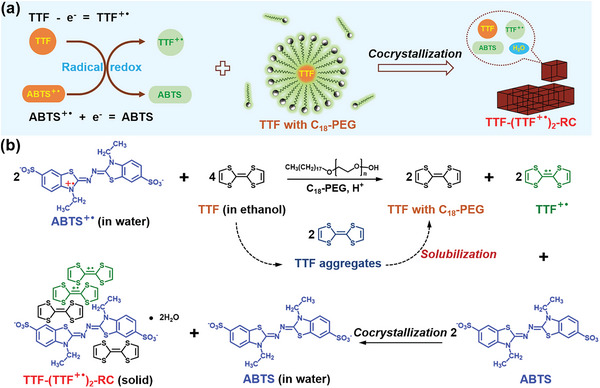
a) Schematic diagram for the combination of radical redox reaction and the solubilization of surfactant for the synthesis of mixed‐valence radical crystal. b) Radical redox reaction between ABTS^+•^ and TTF molecules, and C_18_‐PEG‐assisted synthesis process of TTF‐(TTF^+•^)_2_‐RC.

The stable mixed‐valence radical crystal TTF‐(TTF^+•^)_2_‐RC was synthesized by combining the redox reaction of radicals and the solubilization of surfactants (**Figure** **1**a). Initially, persistent ABTS^+•^ stock solutions were prepared according to our previously reported method (Figures S1 and S2, Supporting Information).^[^ ^10^ ^]^ The excellent electron‐accepting capability of ABTS^+•^ enabled the quantitative one‐electron oxidation with stoichiometric amounts of TTF in aqueous solutions (Figure 1b; Figure S3, Supporting Information). Green color of ABTS^+•^ aqueous solutions instantly turned reddish brown after adding TTF, and two new absorption peaks at 434 and 577 nm appeared, which could be assigned to the oxidation product of TTF, TTF^+•^ (Figure S4, Supporting Information). Electron spin resonance (ESR) spectra showed an intense signal with *g* = ≈2.0081 for TTF^+•^ and a relatively weak signal with *g* = ≈2.0043 for ABTS^+•^, indicating the formation of new unpaired electrons after the oxidation reaction of radicals (Figure S5, Supporting Information). It should be noted that TTF^+•^ was unstable in aqueous solutions even for several hours (Figure S6, Supporting Information), owing to the sensitivity to water and oxygen. Hence, the TTF^+•^ in obtained ABTS/TTF^+•^ system eventually disappeared. Surprisingly, the introduction of surfactants C_18_‐PEG into the ABTS^+•^/TTF system with excess TTF not only increased the clarity of the mixed solutions, but also produced bright red microcrystals, with a length of 30–50 µm and a thickness of ≈5 µm, which could be observed from their optical microscopic, TEM and SEM images (Figures S7–S10, Supporting Information).

To gain a deeper insight into how the surfactant C_18_‐PEG assists the growth of TTF‐(TTF^+•^)_2_‐RC, we added two common surfactants, hexadecyl trimethyl ammonium bromide (CTAB) and sodium dodecyl sulfate (SDS) into same systems, respectively. The results in Figure S11 (Supporting Information) showed that no red microcrystals were produced with the adding of CTAB and SDS. Considering that surfactants mainly played the role of increasing solubility, the solubilization ability of three surfactants to hydrophobic TTF was further investigated. It turned out, although there were little differences in the number of methylene groups at the hydrophobic end compared to C_18_‐PEG, neither CTAB nor SDS could well solubilize hydrophobic TTF molecules (Figure S12, Supporting Information). As a consequence, the possible growth mechanism of TTF‐(TTF^+•^)_2_‐RC was proposed in Figure 1b and Figure S13 (Supporting Information). When TTF contacted with persistent ABTS^+•^, the redox reaction of radicals occurred and two reaction products including hydrophilic ABTS and amphiphilic TTF^+•^ were obtained. With the solubilization of C_18_‐PEG, excess TTF could be readily dispersed and thus easily cocrystallize with ABTS and TTF^+•^ in mixed aqueous solutions. It was worth noting that too low a concentration of C_18_‐PEG would lead to crystalline impurities, and an excessive concentration of C_18_‐PEG could also solubilize targeted crystals and result in a low yield (Figure S14, Supporting Information). Therefore, a suitable mass concentration of C_18_‐PEG (0.83 wt.%) was selected for the large‐scare preparation of TTF‐(TTF^+•^)_2_‐RC.

### 2.2 Single‐Crystal Structure Analysis of the Radical Crystal TTF‐(TTF^+•^)_2_‐RC

**Figure 2:**
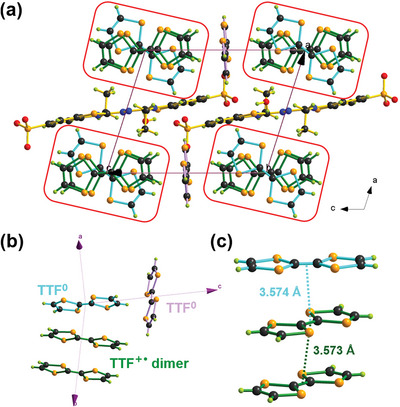
a) Single‐crystal packing of TTF‐(TTF^+•^)_2_‐RC along the *b*‐axis. b) The three‐dimensional structure of four TTF moieties in unit cell. c) The distance between TTF moieties in the TTF dicationic trimer. Color scheme: C, black; S, orange; O, red; N, blue; H, lime.

The structural analysis of TTF‐(TTF^+•^)_2_‐RC was obtained by using SC‐XRD. As shown in **Figure** **2**a and Table S1 (Supporting Information), TTF‐(TTF^+•^)_2_‐RC crystallized in the triclinic *P‐1* space group and comprised four TTF molecules, one ABTS counterion, and two water molecules. The absence of cation/anion contacts in the unit cell suggested that electrostatic interactions were minimal (Figure 2a and Figure S15, Supporting Information). The intermolecular O–H···O hydrogen bonds (2.829 Å, Figure S16, Supporting Information) were formed between ABTS and surrounding water molecules. The stoichiometry of the crystal implied two positive charges for four TTF entirety, which were unevenly distributed in the crystallographic *b* and *c* axes. To obtain more view into the oxidation state for four TTF in TTF‐(TTF^+•^)_2_‐RC, their bond parameters were analyzed in detail (Table S2, Supporting Information). According to their different geometry structures, four TTF molecules were divided into three types: blue, green, and purple one (Figure 2b). For blue and purple TTF, the central C–C bond lengths were 1.334 and 1.353 Å, and average C–S bond lengths were 1.747 and 1.749 Å, respectively. These bond parameters seemed to be more consistent with neutral TTF in works reported elsewhere.^[^ ^17^, ^24^ ^]^ In contrast, two green TTF had an elongated central C–C bond length of 1.385 Å and a shorted average C–S bond length of 1.722 Å, suggesting the one‐electron oxidized state, TTF^+•^.^[^ ^17a^ ^]^

Early study of partially oxidized TTF analogs indicated that changes in the bond length were closely related to the charge residing on the corresponding moiety.^[^ ^25^ ^]^ The charge values q of three types of TTF molecules in TTF‐(TTF^+•^)_2_‐RC calculated based on two empirical formulas were listed in Table S3 (Supporting Information). Expectedly, blue and purple TTF preferred to be electrically neutral, and two green TTF had positive charges +0.83/0.87 that could be considered as TTF^+•^. As shown in Figure S17 (Supporting Information), there were continuous TTF–TTF^+•^–TTF^+•^ trimer structures with short intermolecular distances in b axes, suggesting the existence of strong interactions in TTF‐(TTF^+•^)_2_‐RC. Within the TTF dicationic trimer, two adjacent TTF^+•^ were centrosymmetric with an interplanar separation of 3.573 Å (Figure 2c). Due to a 2.6° angel between TTF^+•^ planes, the distances between atoms of two TTF^+•^ molecules varied from 3.475 to 3.629 Å (Figure S18, Supporting Information), while intermolecular S···S distances were 3.508 and 3.629 Å, shorter than the sum of the van der Waals radii (3.70 Å), indicative of the presence of TTF^+•^ radical *π*‐dimers. Moreover, the coplanar neutral TTF was rotated by 44.9° relative to the TTF^+•^ dimers with an adjacent intermolecular distance of ≈3.574 Å (Figure 2c and Figure S18, Supporting Information), suggesting the existence of strong CT interactions between neutral TTF and adjacent TTF^+•^.

### 2.3 Component Characterizations of the Radical Crystal TTF‐(TTF^+•^)_2_‐RC

**Figure 3:**
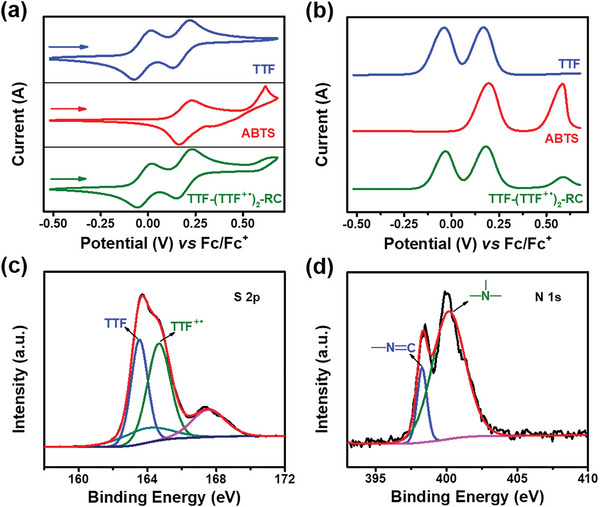
a) Cyclic voltammograms and b) Differential pulse voltammograms of TTF, ABTS, and TTF‐(TTF^+•^)_2_‐RC at 200 mV s^−1^. The experiments were conducted in 0.1 m TBAPF_6_/DMSO electrolyte. The XPS spectra of c) S 2p and d) N 1s of TTF‐(TTF^+•^)_2_‐RC.

The phase purity of TTF‐(TTF^+•^)_2_‐RC was verified by powder X‐ray diffraction (P‐XRD) measurements, where main positions of diffraction peaks were well consistent with the simulated pattern (Figure S19, Supporting Information). To investigate the redox property of TTF‐(TTF^+•^)_2_‐ RC, the liquid state cyclic voltammograms (CVs) and differential pulse voltammograms (DPVs) of TTF, ABTS, and TTF‐(TTF^+•^)_2_‐RC were measured in dimethyl sulfoxide (DMSO) containing 0.1 m tetra‐*n*‐butyl‐ammoniumhexa‐fluorophosphate (TBAPF_6_) electrolyte. As shown in **Figure** **3**a, the CV of TTF revealed two consecutive reversible redox process. The oxidation peak at 0.021 and 0.226 V (vs Fc/Fc^+^) could be assigned to two one‐electron oxidation processes, which were TTF/TTF^+•^ and TTF^+•^/TTF^2+^, respectively. The reversible oxidation peak at 0.231 V (vs Fc/Fc^+^) was assigned to ABTS/ABTS^+•^ redox couple, and the oxidation peak at 0.618 V (vs Fc/Fc^+^) was irreversible due to the instability of ABTS^2+^. Since oxidation peaks of TTF^+•^/TTF^2+^ and ABTS/ABTS^+•^ were very close together and similar, the CV of TTF‐(TTF^+•^)_2_‐RC exhibited three main peaks at 0.019, 0.232, and 0.626 V (vs Fc/Fc^+^) (Figure 3a). These redox processes were more clearly observed in the DPV of TTF, ABTS, and TTF‐(TTF^+•^)_2_‐RC (Figure 3b).

Then, X‐ray photoelectron spectroscopy (XPS) was conducted to confirm the mixed‐valence state in TTF‐(TTF^+•^)_2_‐RC. The S 2p band could be deconvoluted into four bands (Figure 3c), in which two bands at 163.58 and 164.68 eV were unambiguously assigned to neutral TTF and TTF^+•^, respectively.^[^ ^17^, ^26^ ^]^ The other two bands at 164.38 and 167.58 eV were ascribed to the thiazole ring and the sulfonic acid group in ABTS counterion, respectively. Moreover, N 1s multiple‐peak fitting results showed that ABTS diammonium salts had an exclusive band at 401.58 eV belonging to the NH_4_^+^ moiety compared to TTF‐(TTF^+•^)_2_‐RC (Figure 3d; Figure S20, Supporting Information), which suggested that ABTS existed in the form of counterions without diammonium salts in TTF‐(TTF^+•^)_2_‐RC. Both Raman (Figure S21, Supporting Information) and Fourier transform infrared (FTIR) spectra (Figure S22, Supporting Information) of TTF‐(TTF^+•^)_2_‐RC were the sum of main peaks for TTF and ABTS powders, and subtle shift changes might be caused by distinct chemical environments or different oxidation states.

### 2.4 Magnetic Properties of the Radical Crystal TTF‐(TTF^+•^)_2_‐RC

**Figure 4:**
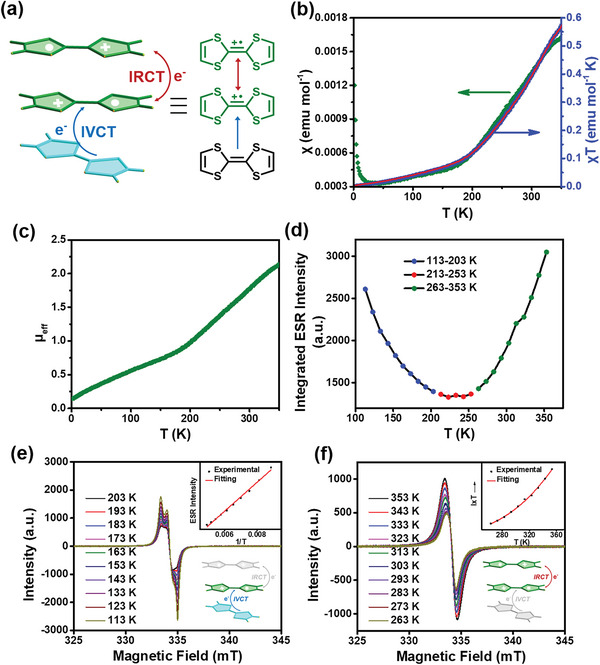
a) Schematic diagram of dual CT interactions in the TTF dicationic trimer. IRCT: Inter‐radical charge transfer; IVCT: Inter‐valence charge transfer. b) Temperature‐dependent plots of the molar magnetic susceptibility, 𝜒 of TTF‐(TTF^+•^)_2_‐RC (green rhombics, left scale), and the 𝜒𝑇 product (blue rhombics, right scale) together with its fit using the Bleaney−Bowers equation (red line). c) Variation of the effective magnetic moments (µ_eff_, in Bohr's magnetons) with temperature for TTF‐(TTF^+•^)_2_‐RC. d) Temperature‐dependent plots of integrated ESR intensities for TTF‐(TTF^+•^)_2_‐RC in the temperature range of 113–353 K. e) Variable‐temperature ESR spectra of TTF‐(TTF^+•^)_2_‐RC in the temperature range of 113–203 K. f) Variable‐temperature ESR spectra of TTF‐(TTF^+•^)_2_‐RC in the temperature range of 263–353 K. Inset: temperature‐dependent plot of IxT (I: Integrated ESR intensity; T: Temperature).

As shown in **Figure** **4**a, short intermolecular distances between TTF moieties within the TTF dicationic trimer facilitated dual CT transitions, including the IVCT between neutral TTF and TTF^+•^, and the IRCT between two TTF^+•^ in dimer. To quantitatively examine the spin state equilibrium and clarify the ground state electronic structures of TTF‐(TTF^+•^)_2_‐RC, superconducting quantum interference device (SQUID) measurements were carried out at the temperature range from 2 to 350 K. As shown in Figure 4b, the molar magnetic susceptibility *χ* of TTF‐(TTF^+•^)_2_‐RC (green points) decreased sharply with a drop in temperature from 350 to 25 K. A plot of the *χ*T products (blue points) versus temperature could be well‐fitted using modified Bleaney−Bowers equations that can describe the magnetic behavior of systems with a pair of interacting spins.^[^ ^11b^ ^]^ Through the calculation of fitting results, the value of 2*J* = −657 ± 26 cm^−1^ was obtained, indicative of the antiferromagnetic coupling of two spins in the TTF dicationic trimers. Furthermore, the effective magnetic moment (µ_eff_) of TTF‐(TTF^+•^)_2_‐RC was 2.14 Bohr magnetons per trimeric unit when the temperature reached 350 K (Figure 4c). This number was ≈75.6% of the theoretical value of 2.83 Bohr magnetons expected for two unpaired electrons (triplet state) per unit, which suggested that a substantial fraction of the electrons in TTF‐(TTF^+•^)_2_‐RC were unpaired at this high temperature.

**Figure 5:**
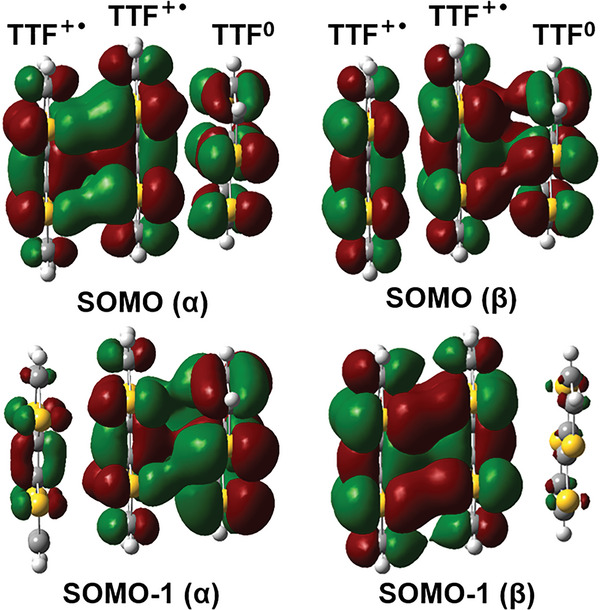
Selected molecular orbitals for the TTF dicationic trimer in TTF‐(TTF^+•^)_2_‐RC.

To further explore the magnetic property, solid‐state ESR spectra of crystalline TTF‐(TTF^+•^)_2_‐RC were measured. The room‐temperature ESR spectrum of crystalline TTF‐(TTF^+•^)_2_‐RC showed a strong signal with g value of 2.0086, which was a characteristic value for TTF^+•^ (Figure S23, Supporting Information).^[^ ^16a^ ^]^ Variable‐temperature ESR (VT‐ESR) measurement of crystalline TTF‐(TTF^+•^)_2_‐RC presented a decreased trend of integrated ESR intensity with the temperature rising from 113 to 203 K (Figure 4d), and showed a linear correlation relative to temperature‐dependent ESR intensity (Figure 4e), similar to those observed in typical monoradicals,^[^ ^27^ ^]^ which was likely due to the IVCT interaction between neutral TTF and adjacent TTF^+•^. However, the trend of integrated ESR intensity became reverse to increase with the temperature rising from 263 to 353 K (Figure 4d,f), revealing the thermally populated triplet state for the TTF dicationic trimer,^[^ ^28^ ^]^ which might be originated from the spin‐spin interaction in the (TTF^+•^)_2_ *π*‐dimer. The IRCT transition between adjacent TTF^+•^, in other words, the spin‐spin interaction between two unpaired electrons in triplet states was predominant at the higher temperature. The peak shape varied from cracking to a single peak also well proved the transformation of electronic spin state. Besides, the negligible difference of integrated ESR intensity with temperature from 213 to 253 K indicated a balance between IVCT and IRCT. Density functional theory (DFT) calculations were further carried out at the UB3LYP/6‐31G(d,p) level for the TTF dicationic trimer (with the geometry extracted from the X‐ray structure of TTF‐(TTF^+•^)_2_‐RC). The calculated results confirmed the open‐shell singlet ground state of this dicationic trimer. The calculated energy gap of open‐shell singlet and triplet was 1.80 kcal mol^−1^, which was close to the estimated value of 1.88 kcal mol^−1^ according to the SQUID measurements. We further calculated the frontier orbitals and corresponding energies of the TTF trimer within the TTF‐(TTF^+•^)_2_‐RC in Figure S24 (Supporting Information). As shown in **Figure** **5**, the calculated singly occupied molecular orbital (SOMO) profiles of the *α* and *β* spins of the TTF dicationic trimer showed significant overlaps, which clearly depicted intermolecular interactions, including IRCT between two adjacent TTF^+•^, and IVCT between neutral TTF and TTF^+•^.

### 2.5 Solid‐State Absorption and Stability of the Radical Crystal TTF‐(TTF^+•^)_2_‐RC

**Figure 6:**
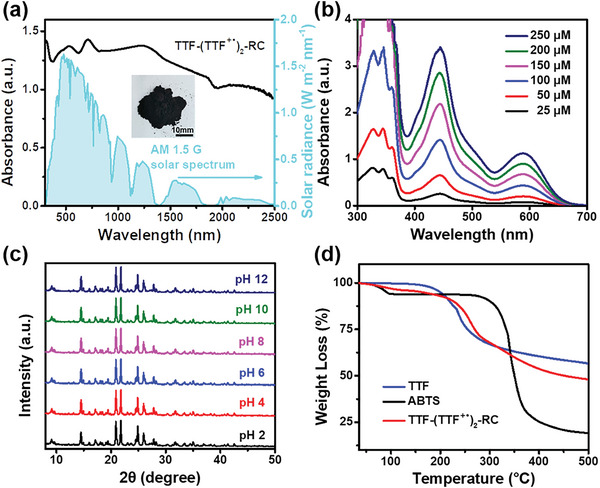
a) Solid‐state absorption spectrum of TTF‐(TTF^+•^)_2_‐RC. Inset: photograph of crystal powders. b) UV‐vis absorption spectra of dissolved TTF‐(TTF^+•^)_2_‐RC with different concentration in DMSO. c) Powder X‐ray diffraction patterns for TTF‐(TTF^+•^)_2_‐RC at different pH aqueous solutions. d) TG spectra of TTF, ABTS, and TTF‐(TTF^+•^)_2_‐RC powders.

The solid‐state UV–vis–NIR absorption spectrum of TTF‐(TTF^+•^)_2_‐RC is shown in **Figure** **6**a. Compared to TTF and ABTS powders, TTF‐(TTF^+•^)_2_‐RC exhibited a strong light absorption over a broad spectral range that effectively covered the full solar spectrum (Figure 6a and Figure S25, Supporting Information). It should also be mentioned that the absorption of TTF‐(TTF^+•^)_2_‐RC seemed to be the sum of IVCT transitions between neutral TTF and TTF^+•^ and IRCT transitions between two TTF^+•^ in dimer. The absorption peak at 534 nm was attributable to localized excitations inside each TTF^+•^ moiety of the dimer. The sharpest absorption peak at 712 nm was attributed to a CT transition and was the most distinctive indication of the intermolecular CT interaction between two TTF^+•^ in the dimer.^[^ ^13^ ^]^ In addition, the broad NIR absorption over 1000 nm was attributable to the IVCT bands between neutral TTF and TTF^+•^.^[^ ^11^, ^17^ ^]^ To further verify the full‐spectrum absorption was due to strong intermolecular interactions, we also measured a series of absorption spectra of dissolved TTF‐(TTF^+•^)_2_‐RC. As shown in Figure 6b, both high and low concentrations of dissolved TTF‐(TTF^+•^)_2_‐RC in DMSO exhibited main absorption bands of TTF^+•^ at 444 and 589 nm while no absorbance over 700 nm.

Most of the radical species were unstable and easily reoxidized when exposed to air or water. To evaluate the acidic/basic stability of TTF‐(TTF^+•^)_2_‐RC, we kept crystalline TTF‐(TTF^+•^)_2_‐RC in aqueous solutions at various pH values for 3 days and used P‐XRD measurements to investigate the stability. The results in Figure 6c showed that TTF‐(TTF^+•^)_2_‐RC retained its crystal structure, indicative of the good stability in acidic/basic environments. Moreover, thermogravimetric (TG) analysis indicated that TTF‐(TTF^+•^)_2_‐RC exhibited good thermal stability, and a slight weight loss below 100 °C could be due to the evaporation of crystal water (Figure 6d). As shown in Figure S26 (Supporting Information), the synchronous TG‐DSC analysis showed that there was no obvious endothermic peak before the sample loses weight and no glass transition or melting peak before decomposition.

### 2.6 Photothermal Performance and Stability of the Radical Crystal TTF‐(TTF^+•^)_2_‐RC

With efficient absorption across the full spectrum and superior stability, TTF‐(TTF^+•^)_2_‐RC was expected to be an excellent organic photothermal material for converting both NIR light and solar energy to heat. To systematically evaluate the photothermal property of TTF‐(TTF^+•^)_2_‐RC, NIR laser, and simulated solar light were used to investigate its photothermal performance. First, TTF‐(TTF^+•^)_2_‐RC powders were put on a quartz glass substrate and irradiated by a 1064 nm laser to determine its NIR photothermal conversion capability. The surface temperatures of TTF‐(TTF^+•^)_2_‐RC sharply increased under laser irradiation with different power densities, and could rise up to 130 °C when the power density was 0.6 W cm^−2^ (Figure S27, Supporting Information). Additionally, there was no obvious change in photothermal effect after five on/off laser cycles (Figure S28, Supporting Information), which indicated the excellent photothermal stability of TTF‐(TTF^+•^)_2_‐RC. The photothermal conversion efficiency (PCE) of TTF‐(TTF^+•^)_2_‐RC was calculated to be 62.9% according to the cooling curve (Figure S29, Supporting Information), demonstrating appreciable efficiency compared to other reported organic photothermal materials (Table S4, Supporting Information).^[^ ^10^, ^15^, ^29^ ^]^

**Figure 7:**
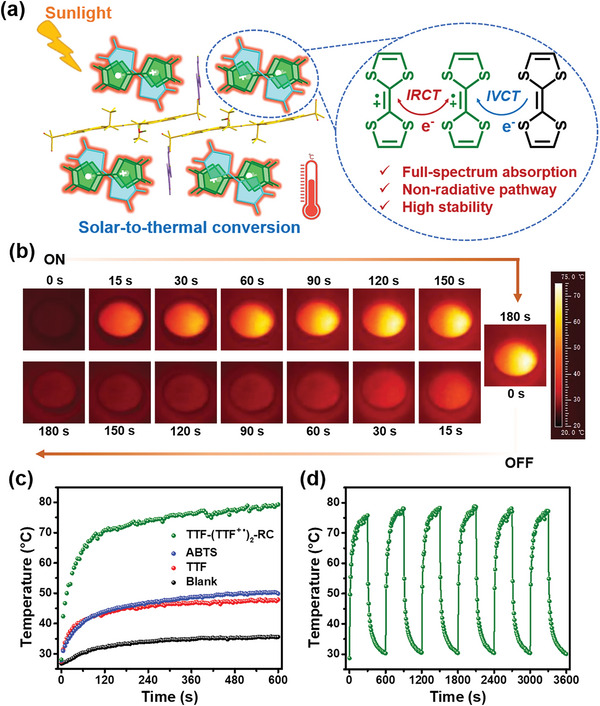
a) Illustration of the high‐efficient solar‐to‐thermal conversion of the TTF‐(TTF^+•^)_2_‐RC. b) IR thermal images of TTF‐(TTF^+•^)_2_‐RC powders under one‐sun irradiation and the cooling process. c) Photothermal conversion curves of TTF‐(TTF^+•^)_2_‐RC, ABTS, and TTF powders with equality mass under one‐sun irradiation. d) Photothermal cycling curves of TTF‐(TTF^+•^)_2_‐RC powder under one‐sun illumination.

We then evaluated the solar photothermal conversion ability of TTF‐(TTF^+•^)_2_‐RC with an AM 1.5 G simulated solar light (**Figure** **7**a). As shown in Figure 7b, TTF‐(TTF^+•^)_2_‐RC had a fast response to light and the surface temperature increased by 46.6 °C within 180 s under one‐sun illumination (0.1 W cm^−2^). The TTF‐(TTF^+•^)_2_‐RC sample showed a quick temperature raise and reached a quasi‐steady plateau of 79.3 °C in 600 s, while ABTS and TTF powders could only rise up to 50.3 and 47.9 °C, respectively (Figure 7c). The superior photothermal effect of TTF‐(TTF^+•^)_2_‐RC was mainly attributed to the sufficient solar absorption due to dual CT transitions. Furthermore, the strong electron delocalization and dual IVCT and IRCT interactions in TTF‐(TTF^+•^)_2_‐RC facilitated the photothermal conversion by suppressing the photoemission process. On the other hand, the outstanding photothermal stability of TTF‐(TTF^+•^)_2_‐RC was also in favor of solar‐thermal conversion, which was examined by six on/off sunlight cycles (Figure 7d). The XPS (Figure S30, Supporting Information) and PXRD (Figure S31, Supporting Information) results after illuminating 1 h both further validated its high photo and photothermal stability. All photothermal results above supported the perception that TTF‐(TTF^+•^)_2_‐RC could be well applied in photothermal therapy, photothermal catalysis, and solar energy utilization.

## 3 Conclusion

The combination of redox reaction of radicals and solubilization of surfactants enabled the successful synthesis of dual CT‐containing stable mixed‐valence radical crystal TTF‐(TTF^+•^)_2_‐RC. Short intermolecular distances between adjacent TTF moiety within TTF‐(TTF^+•^)_2_‐RC could facilitate both IVCT between neutral TTF and TTF^+•^ and IRCT between two TTF^+•^ in dimer. The coexistence of dual CT transitions in TTF‐(TTF^+•^)_2_‐RC were responsible for the unprecedented temperature‐dependent magnetic property and intense full‐spectrum solar absorption. Under NIR laser and one‐sun illumination, TTF‐(TTF^+•^)_2_‐RC exhibited remarkable NIR photothermal property with the PCE of 62.9%, and solar photothermal property, an increase of 46.6 °C within 180 s, respectively. This work would offer new insights for designing radical‐based functional materials through dual or multiple CT engineering, and pave the way of developing new strategy to construct high‐efficiency organic photothermal materials.

## 4 Experimental Section

Experimental details, including synthesis of TTF‐(TTF^+•^)_2_‐RC, characterizations, charge calculations, SQUID studies, ESR studies, and photothermal properties of the stable mixed‐valence radical crystals are listed in the Supporting Information.

[CCDC 2224720 contains the supplementary crystallographic data for this paper. These data can be obtained free of charge from The Cambridge Crystallographic Data Centre via www.ccdc.cam.ac.uk/data_request/cif.]

## Conflict of Interest

The authors declare no conflict of interest.

## Supporting information

Supporting Information

## Data Availability

The data that support the findings of this study are available from the corresponding author upon reasonable request.
