# Supplementary material for: Dual Charge Transfer Generated from Stable Mixed‐Valence Radical Crystals for Boosting Solar‐to‐Thermal Conversion
Source: Adv Sci (Weinh). 2023 May 5;10(21):2300980. doi: 10.1002/advs.202300980 (PMC10375089; doi:10.1002/advs.202300980)
Supplement: Supplementary file 1 — Supporting Information [file ADVS-10-2300980-s001.pdf]

ADVANCED SCIENCE
Open Access

Supporting Information

for *Adv. Sci.*, DOI 10.1002/advs.202300980

Dual Charge Transfer Generated from Stable Mixed-Valence Radical Crystals for Boosting Solar-to-Thermal Conversion

*Jieqiong Xu, Jing Guo, Shengkai Li, Yanxia Yang, Weiming Lai, Phouphien Keoingthong, Shen Wang, Liang Zhang, Qian Dong, Zebing Zeng and Zhuo Chen**

Supporting Information

Dual Charge Transfer Generated from Stable Mixed-Valence Radical Crystals for Boosting Solar-to-Thermal Conversion

*Jieqiong Xu,[#] Jing Guo,[#] Shengkai Li, Yanxia Yang, Weiming Lai, Phouphien Keoingthong, Shen Wang, Liang Zhang, Qian Dong, Zebing Zeng, and Zhuo Chen**

J Xu, S Li, Y Yang, P Keoingthong, S Wang, L Zhang, Q Dong, Prof. Z Chen

Molecular Science and Biomedicine Laboratory (MBL), State Key Laboratory of Chemo/Biosensing and Chemometrics, College of Chemistry and Chemical Engineering, College of Biology, Aptamer Engineering Center of Hunan Province, Hunan University, Changsha, 410082, China.

E-mail: zhuochen@hnu.edu.cn

J Guo, W Lai, Prof. Z Zeng

State Key Laboratory of Chemo/Biosensing and Chemometrics, College of Chemistry and Chemical Engineering, College of Biology, Hunan University, Changsha, Hunan, 410082, China.

# These authors contributed equally to this paper.

Table of Contents

1. **Section S1. Materials.**
2. **Section S2. Apparatus.**
3. **Section S3. Preparation of TTF-($TTF^{+•}$)$_2$-RC radical crystals.**
4. **Section S4. CV measurements.**
5. **Section S5. Charge calculations.**
6. **Section S6. SQUID measurements.**
7. **Section S7. ESR measurements.**
8. **Section S8. Theoretical calculations.**
9. **Section S9. Photothermal conversion properties measurement.**
10. **Section S10. Calculation of photothermal conversion efficiency.**
11. **Figure S1. Nanozyme-assisted single-electron oxidation reaction of ABTS.**
12. **Figure S2. UV-vis-NIR absorption spectra of ABTS and single-electron oxidized $ABTS^{+•}$ in aqueous solution.**
13. **Figure S3. Radical redox reaction between TTF and $ABTS^{+•}$.**
14. **Figure S4. UV-vis-NIR absorption spectra of $ABTS^{+•}$ and $TTF^{+•}$ in aqueous solutions.**
15. **Figure S5. ESR spectra of $ABTS^{+•}$ and $TTF^{+•}$ in aqueous solutions.**
16. **Figure S6. The instability of $TTF^{+•}$ in aqueous solutions.**
17. **Figure S7. Photographs of TTF/$ABTS^{+•}$ system with and without C_{18}-PEG.**
18. **Figure S8. Optical micrographs of TTF-($TTF^{+•}$)$_2$-RC.**
19. **Figure S9. TEM images of TTF-($TTF^{+•}$)$_2$-RC.**
20. **Figure S10. SEM images of TTF-($TTF^{+•}$)$_2$-RC.**
21. **Figure S11. Optical micrographs of the precipitate produced with the adding of C_{18}-PEG, CTAB and SDS, respectively.**
22. **Figure S12. Photographs of solubilization effects of TTF in water with the adding of CTAB, C_{18}-PEG and SDS surfactants.**
23. **Figure S13. Proposed schematic diagrams of surfactant-assisted growth TTF-($TTF^{+•}$)$_2$-RC.**
24. **Figure S14. Optical micrographs of the precipitate produced by adding various mass concentration of C_{18}-PEG.**
25. **Figure S15. Crystal packing of TTF-($TTF^{+•}$)$_2$-RC along the a-axis and c-axis.**
26. **Figure S16. The hydrogen bond interaction between ABTS and H_2O molecules in TTF-($TTF^{+•}$)$_2$-RC.**

27. Figure S17. Crystal packing of TTF moieties in TTF-($TTF^{+\bullet}$)$_2$-RC along the a-axis.

28. Figure S18. The geometry of the $TTF^{+\bullet}$ and neutral TTF in the dicationic trimer.

29. Figure S19. XRD spectra of experimental and calculated TTF-($TTF^{+\bullet}$)$_2$-RC.

30. Figure S20. The XPS spectra of N 1s of ABTS powders.

31. Figure S21. Raman spectra of TTF, ABTS, and TTF-($TTF^{+\bullet}$)$_2$-RC.

32. Figure S22. FTIR spectra of TTF, ABTS, and TTF-($TTF^{+\bullet}$)$_2$-RC.

33. Figure S23. ESR spectra of crystalline TTF-($TTF^{+\bullet}$)$_2$-RC.

33. Figure S24. Frontier orbitals and its energies of TTF dicationic trimer in TTF-($TTF^{+\bullet}$)$_2$-RC.

34. Figure S25. Normalized solid-state absorption spectra of TTF and ABTS.

35. Figure S26. TG-DSC analysis of crystalline TTF-($TTF^{+\bullet}$)$_2$-RC.

36. Figure S27. Photothermal heating curves of TTF-($TTF^{+\bullet}$)$_2$-RC powders under the irradiation of 1064 nm laser with different power densities.

36. Figure S28. Photothermal cyclic curve of TTF-($TTF^{+\bullet}$)$_2$-RC powders under the irradiation of 1064 nm laser.

37. Figure S29. The cooling curve of TTF-($TTF^{+\bullet}$)$_2$-RC powders after the irradiation of 1064 nm laser and its corresponding time-Inθ linear curve.

38. Figure S30. The XPS spectra of S 2p and N 1s of TTF-($TTF^{+\bullet}$)$_2$-RC before and after 1 h irradiation.

39. Figure S31. The P-XRD patterns of TTF-($TTF^{+\bullet}$)$_2$-RC before and after 1 h irradiation.

40. Table S1. Crystal data and structure refinement for TTF-($TTF^{+\bullet}$)$_2$-RC.

41. Table S2. Bond length (Å) of TTF moieties in TTF-($TTF^{+\bullet}$)$_2$-RC.

42. Table S3. Average bond length and calculated charges of TTF moieties in TTF-($TTF^{+\bullet}$)$_2$-RC.

43. Table S4. Photothermal conversion efficiency for reported organic crystals materials.

44. References.

Section S1. Materials

2,2'-azino-bis (3-ethylbenzothiazoline-6-sulfonic acid) (ABTS), Tetrathiafulvalene (TTF), polyoxyethylene (100) stearyl ether ($C_{18}H_{37}(OCH_2CH_2)_nOH$, n~100) ($C_{18}$-PEG), Hexadecyl trimethyl ammonium Bromide (CTAB), Sodium dodecyl sulfate (SDS), Tetra-n-butyl-ammoniumhexa-fluorophosphate ($TBAPF_6$), Ferrocene (Fc), were purchased from Sigma-Aldrich and used without further purification. All other chemicals of analytical reagent grade were purchased from Changsha Chemical Reagents Company (Changsha, China) and used without further purification. Double-distilled water (resistivity ≥ 18.2 MΩ cm) was used throughout the experiments.

Section S2. Apparatus

The crystal morphology was observed by a microscope with 40× objective lens (OLYMPUS IX71) and scanning electron microscopy (SEM) with a FEI QuANTA 200 instrument (FEI, CR). The single crystal structure was obtained on an X-ray diffractometer (XRD, Bruker APEX-II CCD) with Ga Kα radiation at low temperature. The powder X-ray diffraction (PXRD) spectra were collected from a UltimaIV X-ray diffractometer over the range 8-50° in 5°/min steps over 10 min. The X-ray photoelectron spectroscopy (XPS) was recorded in a Thermo Scientific K-Alpha instrument. And the binding energies from the spectra were calibrated against the C 1s peak located at 284.8 eV. The Raman spectra were recorded on a Renishaw Raman imaging microscope system (Invia-reflex) with a 633 nm laser excitation. The Fourier Transform infrared spectroscopy (FTIR) spectra were performed on a Shimadzu Plastic Analyzer. The thermogravimetry (TG) spectra were carried out using a STA7200 analyzer with a heating rate of 10 K/min using Ar as the protective gas. The UV-vis-NIR absorption spectra were recorded in the range of 200-2500 nm with a Shimadzu UV 3600 spectrophotometer (Lambda750).

Section S3. Preparation of TTF-$(TTF^{+\bullet})_2$-RC radical crystals

First step, the persistent $ABTS^{+\bullet}$ solution was prepared by nanozyme-assisted single-electron oxidation reaction according our previous method.[S1] Then, 16 mL of the obtained $ABTS^{+\bullet}$ solution and 1.5 mL of the C_{18}-PEG surfactant (10 wt. %) were added into a glass vial. Finally, added 0.6 mL of the TTF (20 mM in ethanol) into it and mixed uniformly. After

4 h growth at 16 °C, a bright red precipitate of TTF-($TTF^{+\bullet}$)$_2$-RC blanketed the bottom of the glass vial. TTF-($TTF^{+\bullet}$)$_2$-RC powders were obtained by collecting these precipitates and then freeze-drying.

Section S4. CV measurements

The cyclic voltammetry (CV) was performed on a Chenhua 650D electrochemical using a three-electrode cell with a glass carbon working electrode, a platinum wire counter electrode, and an Ag/AgCl reference electrode in dimethyl sulfoxide (DMSO) solvent containing the electrolyte $TBAPF_6$ (0.1 M). The ferrocene/ferrocenium redox couple was used as an external reference.

Section S5. Charge calculations

According to previous reports, the two empirical formulas for charge calculations were provided in the following:

$$\partial = (b + c) - (a + d)$$

$$q = A - B \times \partial \qquad (1)$$

$$q_1 = -15.55 + 20.42({}^{a}/_{b})$$

$$q_2 = 4.490 + 10.748(a - b)$$

$$q = (q_1 + q_2)/2 \qquad (2)$$

where A = 6.3362 and B = 7.5911.

Section S6. SQUID measurements

The magnetic susceptibility data were measured using a Quantum DesignXL5 SQUID magnetometer in a 1000 Oe magnetic field in the temperature range from 2 to 350 K in 2 K/min speeds. The Bleaney−Bowers equations that can describe the magnetic behavior of systems with a pair of interacting spins was as follow:

$$\chi T = \frac{2Ng^2\beta^2}{k\left[3 + exp\left(-\frac{2J}{kT}\right)\right]}(1-\rho) + \frac{Ng^2\beta^2}{2k}\rho + (\mathrm{TIP})T$$

where N was the Avogadro number, g was a g tensor, β was a Bohr magneton, ρ was a mole fraction of a paramagnetic impurity, and TIP represented the temperature independent paramagnetism.

The μeff values were calculated at each temperature from the experimental molar magnetic susceptibilities (obtained by SQUID measurements) as $\mu_{eff} = 2.8279 \times (\chi T)$^0.5 (see: Drago, R.S. Physical methods in chemistry, Ch 11. W.B. Sanders Co, Philadelphia, 1997.)[S2]

Section S7. ESR measurements

Electron spin resonance (ESR) measurements were performed on a JES-FA 200 spectrometer. The spectra of aqueous solutions were measured at room temperature. The various temperature ESR spectra of TTF-$(TTF^{+\bullet})_2$-RC powders were measured from 113 K to 353 K at 10 K steps in a nitrogen atmosphere.

Section S8. Theoretical calculations

Density functional theory (DFT) and time-dependent DFT (TD-DFT) simulations were performed using the Gaussian 09 software package. The energy and geometry of the TTF dicationic trimer within TTF-$(TTF^{+\bullet})_2$-RC were calculated at the B3LYP/6-31G(d,p) level based on the crystal structure.

Section S9. Photothermal conversion properties measurement

Firstly, TTF-$(TTF^{+\bullet})_2$-RC powders were put on quartz glass and pressed into a film. A 1064 nm fiber-coupled laser (MIL-H-1064-2W-19110362, Changchun New Industries Optoelectronics Tech. Co., Ltd, China) was used to study the NIR photothermal conversion performance of TTF-$(TTF^{+\bullet})_2$-RC. Secondly, about 300 mg TTF-$(TTF^{+\bullet})_2$-RC powders were weighted into a glass culture dish. A solar simulator with an optical filter for the standard AM 1.5 G spectrum (PLS-SXE300DUV) was used to study the solar photothermal conversion performance of TTF-$(TTF^{+\bullet})_2$-RC. The corresponding temperature changes were measured by an IR thermal camera (FOTRIC 365).

Section S10. Calculation of photothermal conversion efficiency

The NIR photothermal conversion efficiency of TTF-$(TTF^{+\bullet})_2$-RC was determined according the previous methods.[S3] Details were as follows:

Based on the total energy balance for this system:

$$\sum_i m_i C_{pi} \frac{dT}{dt} = Q_s - Q_{loss}$$

where m_i (0.4569 g) and C_{pi} (0.8 J/(g.°C)) are the mass and heat capacity of system components (cocrystal powders and quartz glass), respectively. Q_s is the photothermal heat energy input by irradiating NIR laser to samples, and Q_{loss} is thermal energy lost to the surroundings. When the temperature is maximum, the system is in balance.

$$Q_s = Q_{loss} = hS\Delta T_{max}$$

where h is heat transfer coefficient, S is the surface area of the container, ΔT_{max} is the maximum temperature change. The photothermal conversion efficiency η is calculated from the following equation:

$$\eta = \frac{hS\Delta T_{max}}{I(1 - 10^{-A_{1064}})}$$

where I is the laser power (0.6 W cm^{-2}) and A_{1064} is the absorbance of the samples at the wavelength of 1064 nm (1.3532).

In order to obtain the hS, a dimensionless driving force temperature, θ is introduced as follows:

$$\theta = \frac{T - T_{surr}}{T_{max} - T_{surr}}$$

where T is the temperature of sample, T_{max} is the maximum temperature of the cooling curve (132.1 °C), and T_{surr} is the initial surrounding temperature (22.0 °C).

The sample system time constant τ_s

$$\tau_s = \frac{\sum_i m_i C_{p,i}}{hS}$$

thus $\frac{d\theta}{dt} = \frac{1}{\tau_s} \frac{Q_s}{hS\Delta T_{max}} - \frac{\theta}{\tau_s}$

when the laser is off, $Q_s = 0$, therefore $\frac{d\theta}{dt} = -\frac{\theta}{\tau_s}$, and $t = -\tau_s \ln \theta$

so hS could be calculated from the slope of cooling time vs $\ln \theta$. Therefore, τ_s is 111.48 s (**Fig. S29**) and the photothermal conversion efficiency η is 62.9%.

Supplementary Figures and Tables:

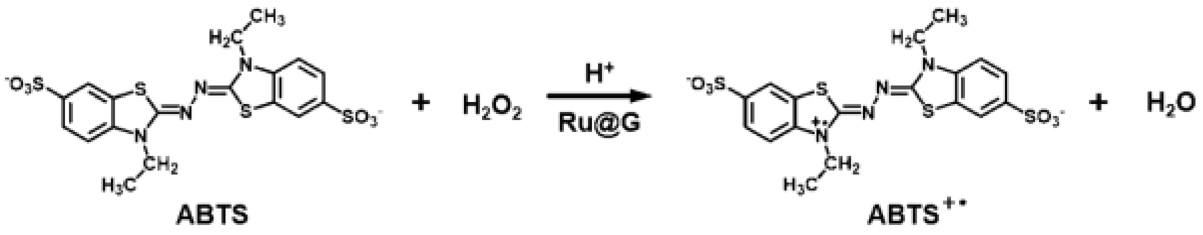

Figure S1. Nanozyme-assisted single-electron oxidation reaction of ABTS.

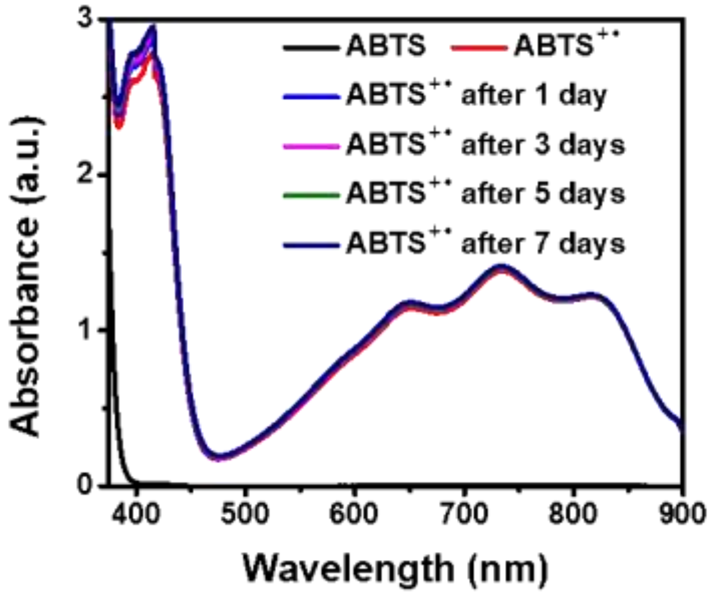

Figure S2. UV-vis-NIR absorption spectra of ABTS and single-electron oxidized ABTS$^{+\bullet}$ in aqueous solution.

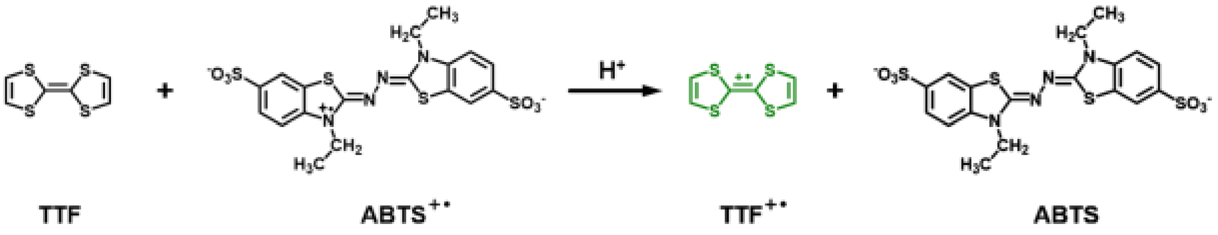

Figure S3. Radical redox reaction between TTF and ABTS$^{+\bullet}$.

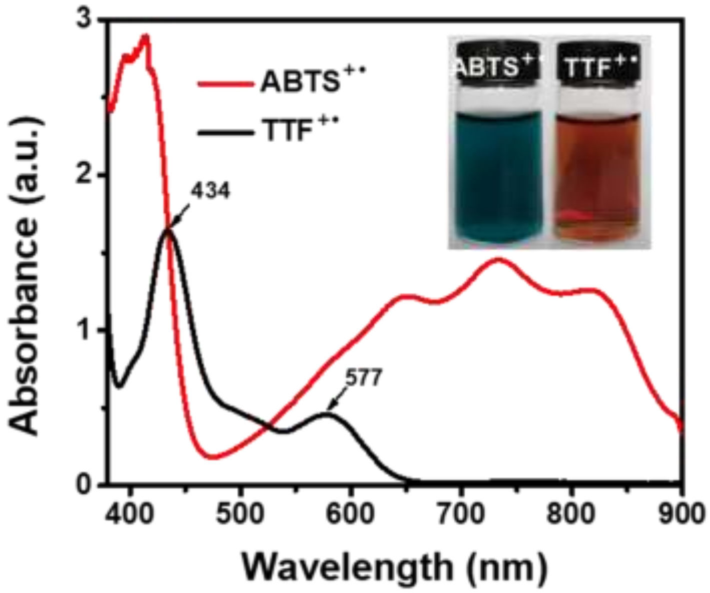

Figure S4. UV-vis-NIR absorption spectra of $ABTS^{+\bullet}$ and $TTF^{+\bullet}$ in aqueous solutions. Insets: photographs of $ABTS^{+\bullet}$ and $TTF^{+\bullet}$ in aqueous solutions.

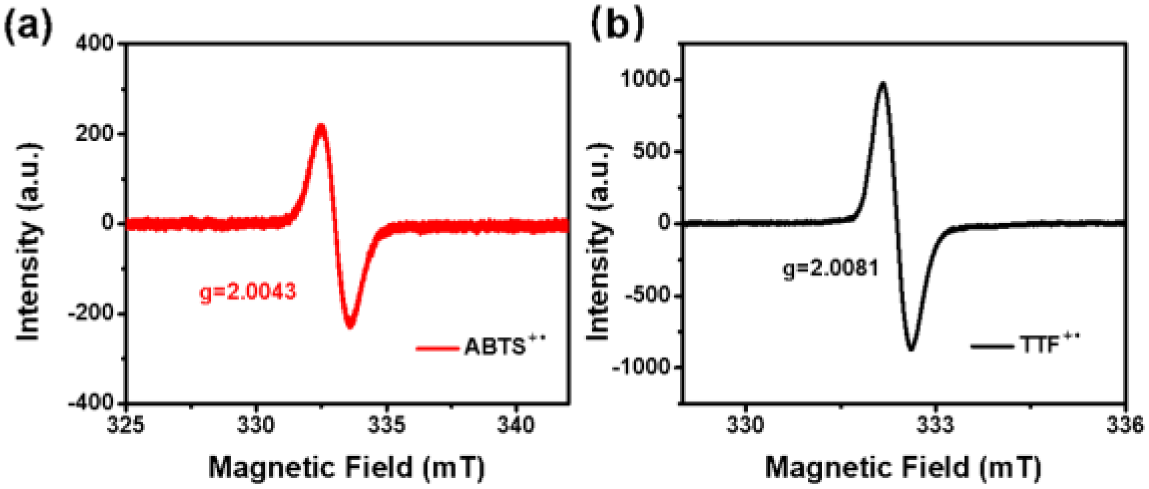

Figure S5. ESR spectra of (a) $ABTS^{+\bullet}$ and (b) $TTF^{+\bullet}$ in aqueous solutions.

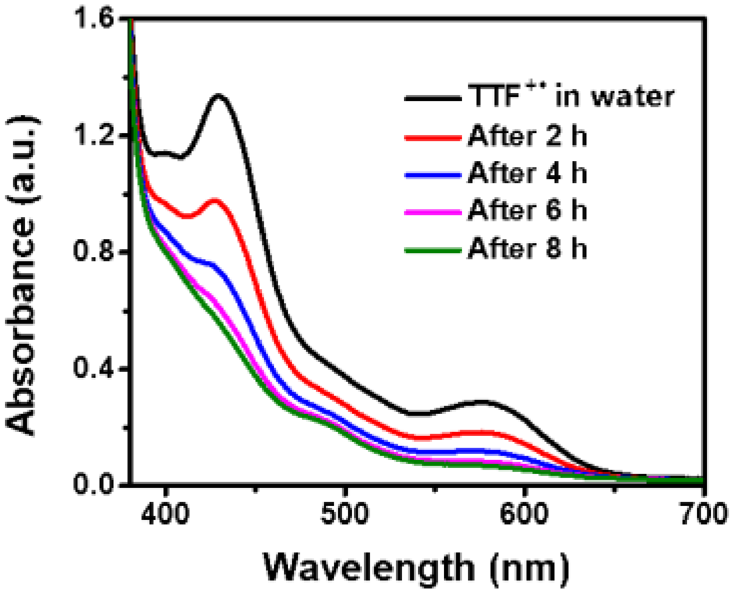

Figure S6. The instability of $TTF^{+\bullet}$ in aqueous solutions.

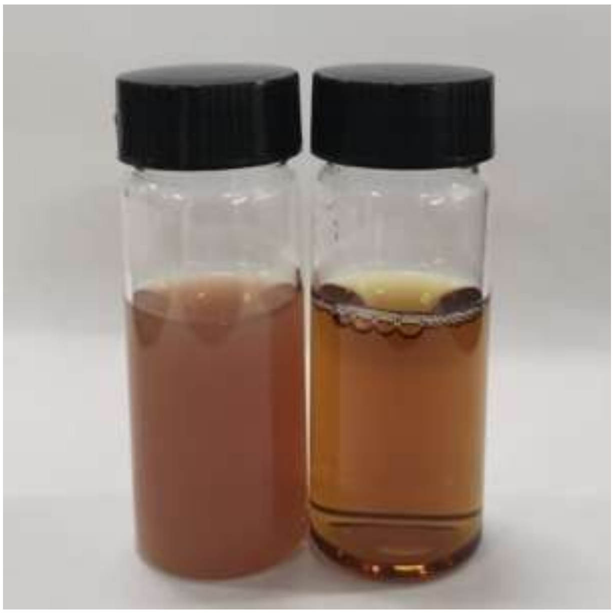

Figure S7. Photographs of TTF/$ABTS^{+\bullet}$ system with (right) and without (left) C_{18}-PEG.

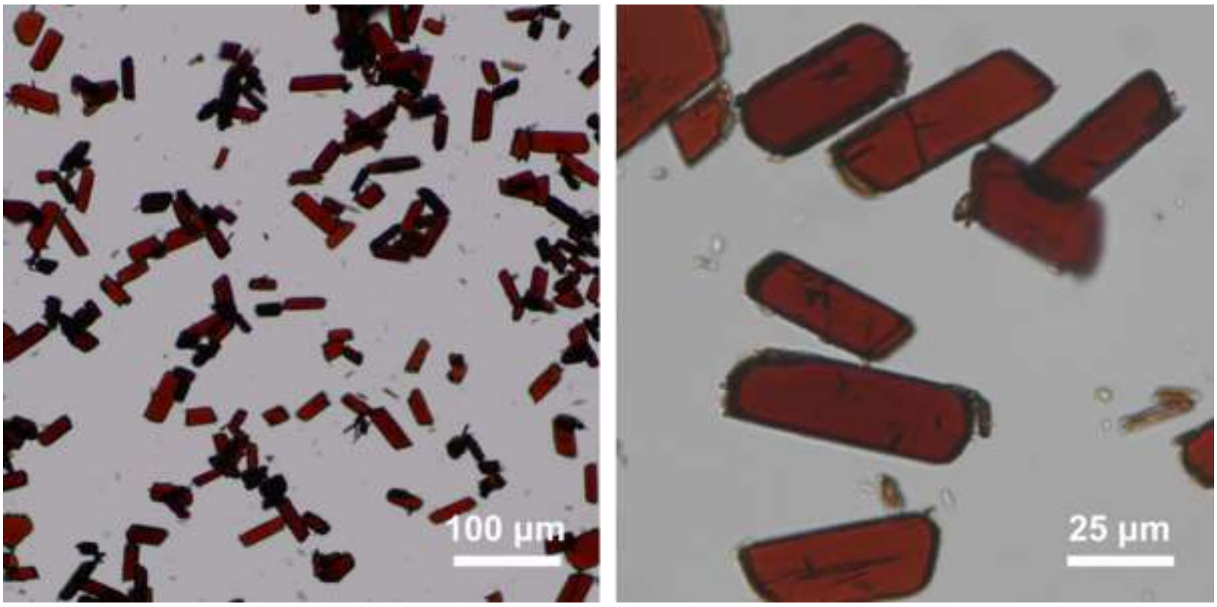

Figure S8. Optical micrographs of TTF-$(TTF^{+\bullet})_2$-RC.

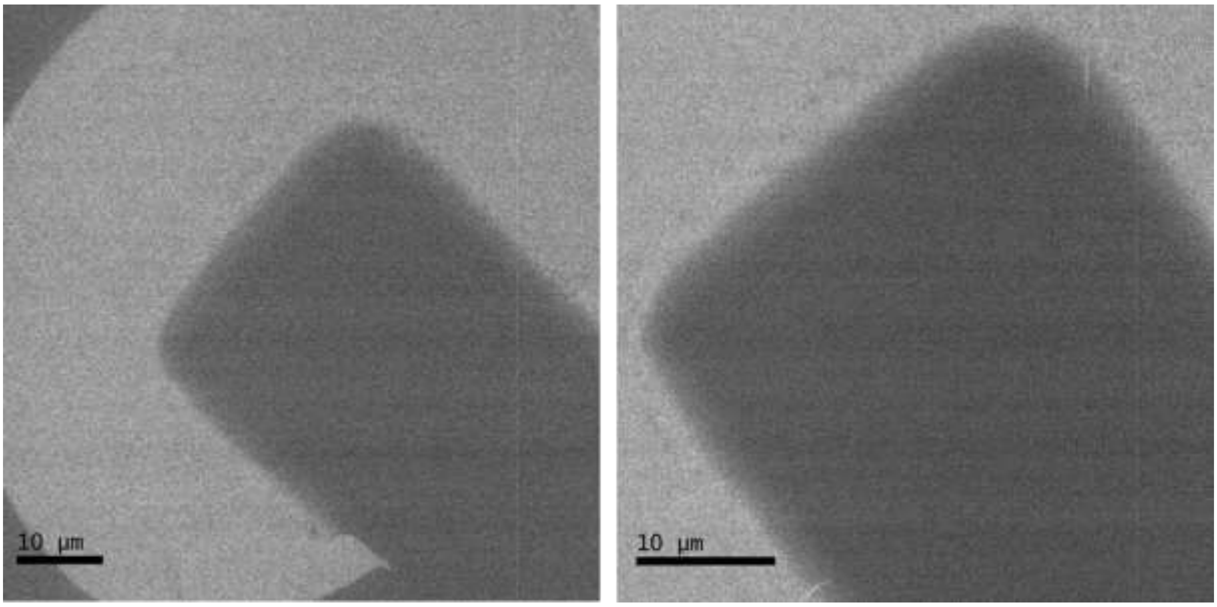

Figure S9. TEM images of TTF-$(TTF^{+\bullet})_2$-RC.

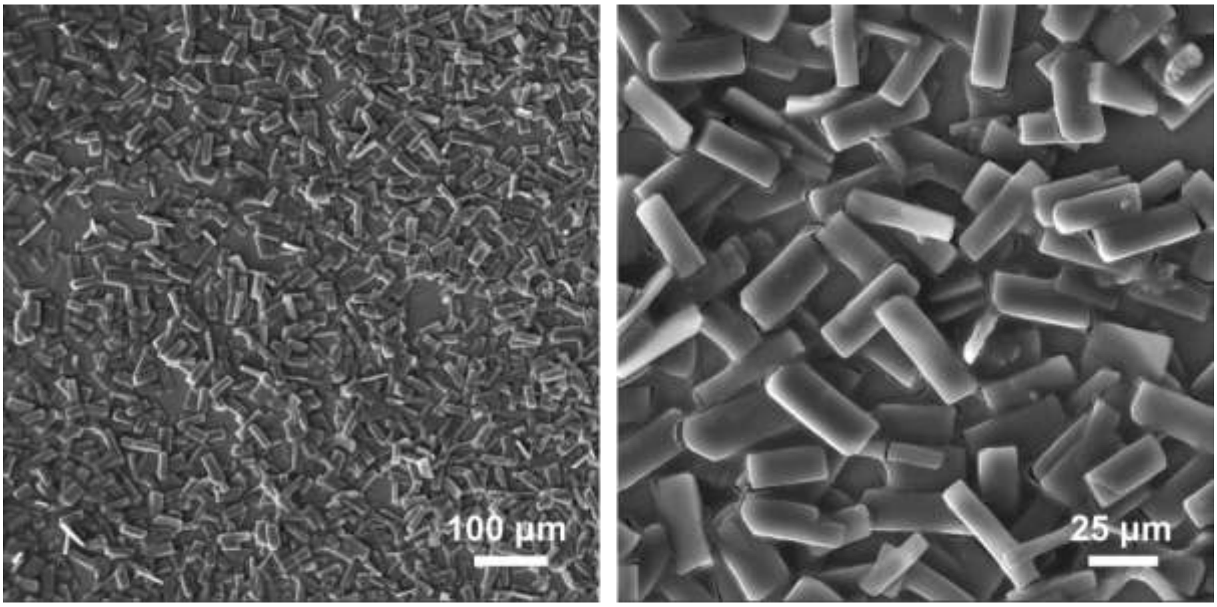

Figure S10. SEM images of TTF-($TTF^{+\bullet}$)$_2$-RC.

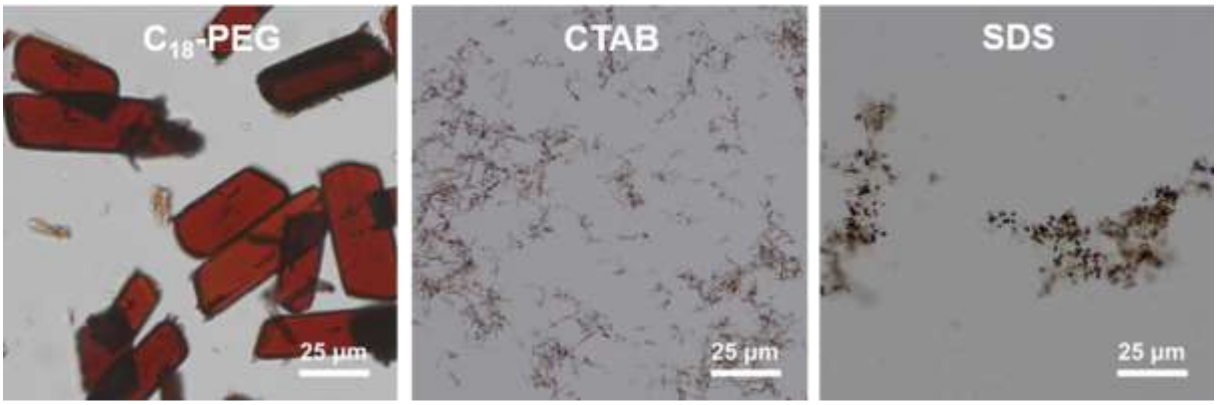

Figure S11. Optical micrographs of the precipitate produced with the adding of C_{18}-PEG, CTAB and SDS, respectively.

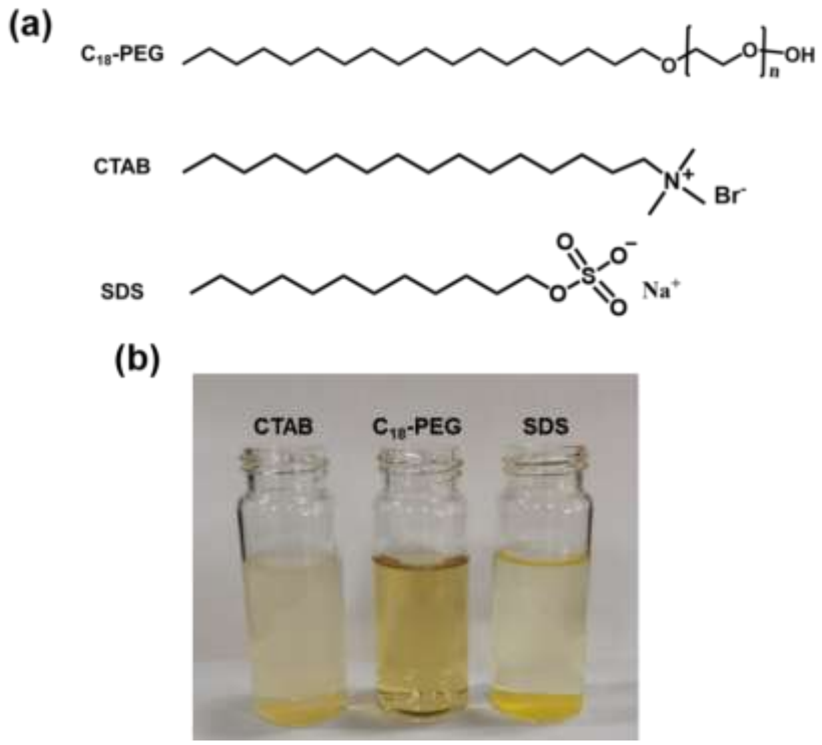

Figure S12. a) Molecular structures of three surfactants including C_{18}-PEG, CTAB and SDS. b) Photographs of solubilization effects of TTF in water with the adding of CTAB, C_{18}-PEG and SDS surfactants.

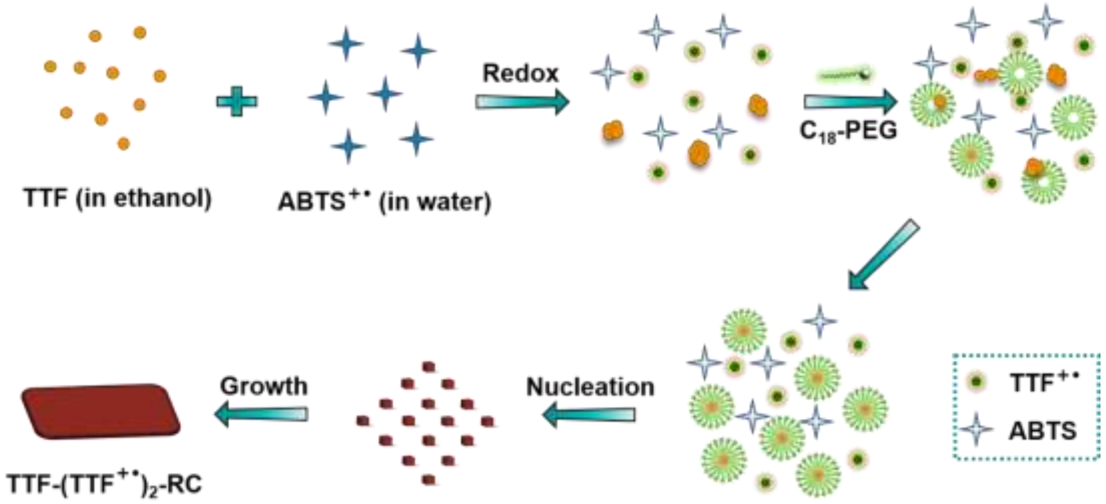

Figure S13. Proposed schematic diagrams of surfactant-assisted growth TTF-($TTF^{+\bullet}$)$_2$-RC.

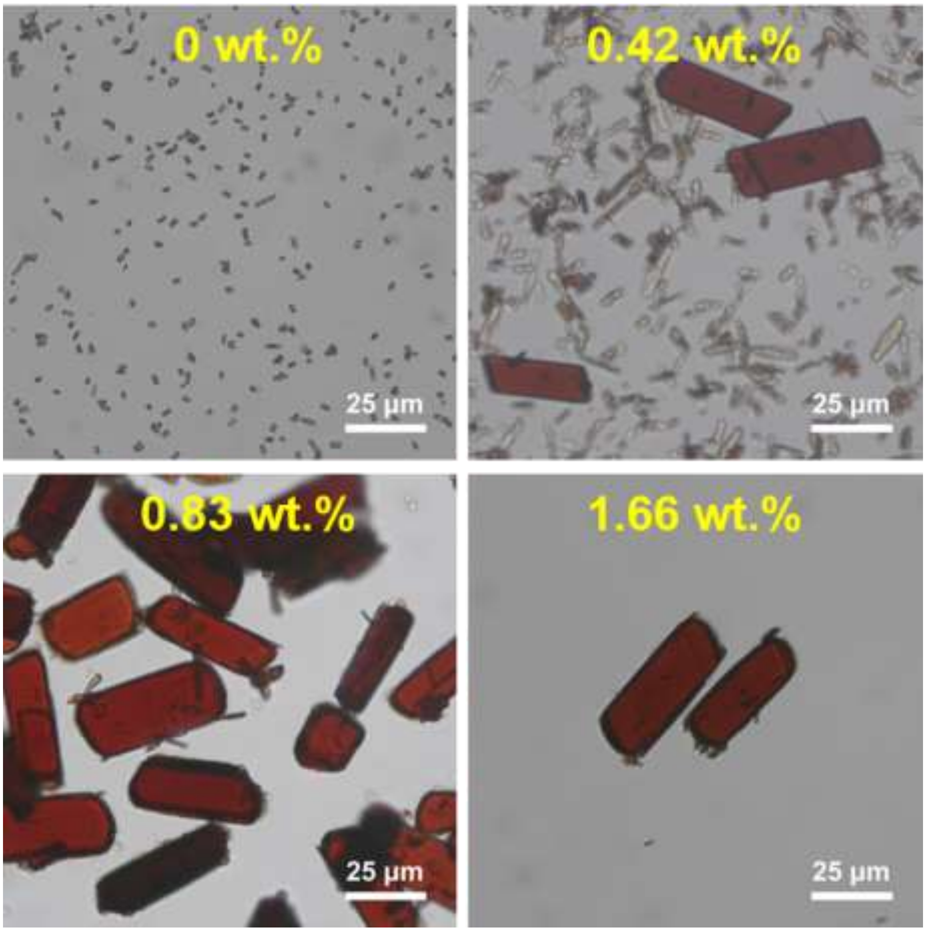

Figure S14. Optical micrographs of the precipitate produced by adding various mass concentration of C_{18}-PEG.

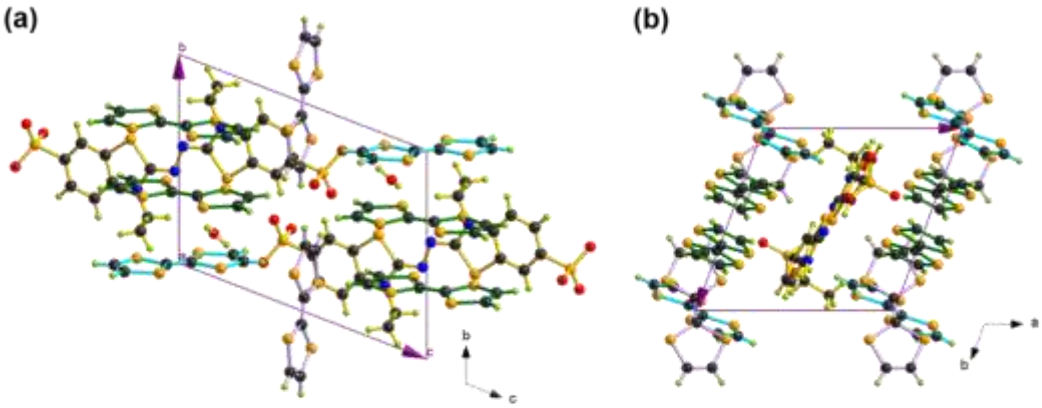

Figure S15. Crystal packing of TTF-(TTF$^{+\bullet}$)$_2$-RC along the a) a-axis and b) c-axis.

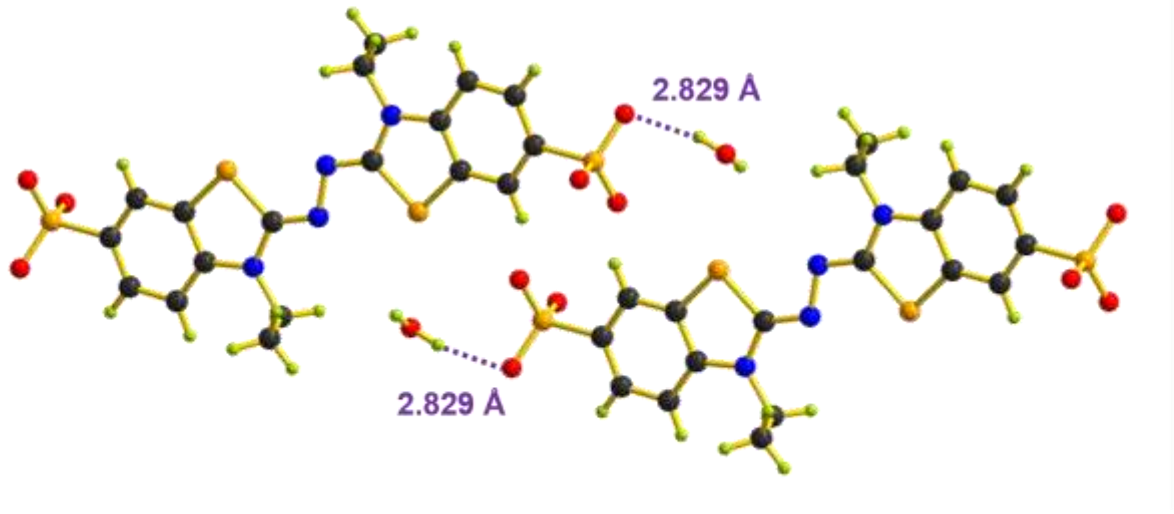

Figure S16. The hydrogen bond interaction between ABTS and H_2O molecules in TTF-$(TTF^{+\bullet})_2$-RC.

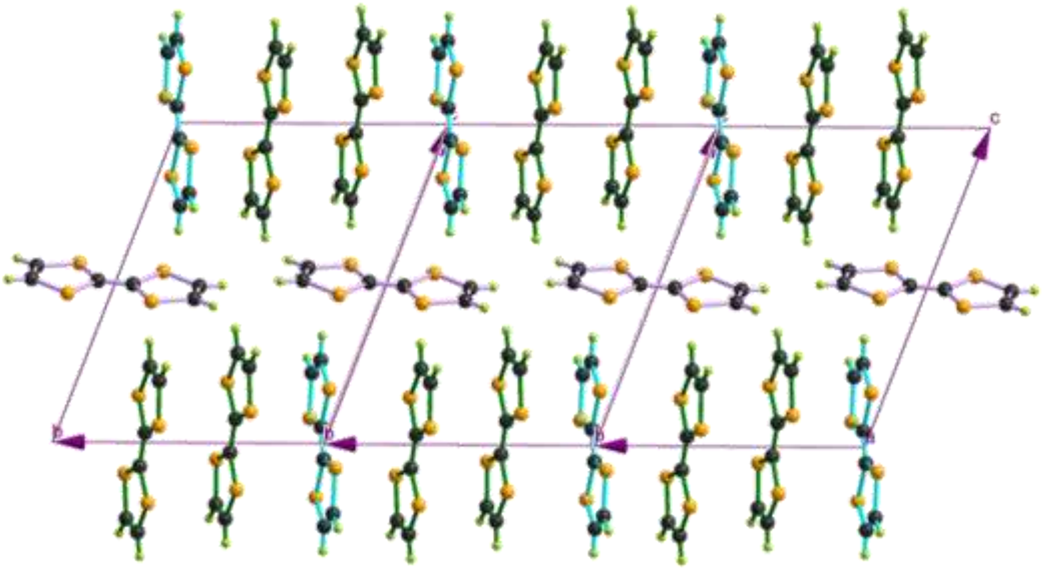

Figure S17. Crystal packing of TTF moieties in TTF-$(TTF^{+\bullet})_2$-RC along the a-axis.

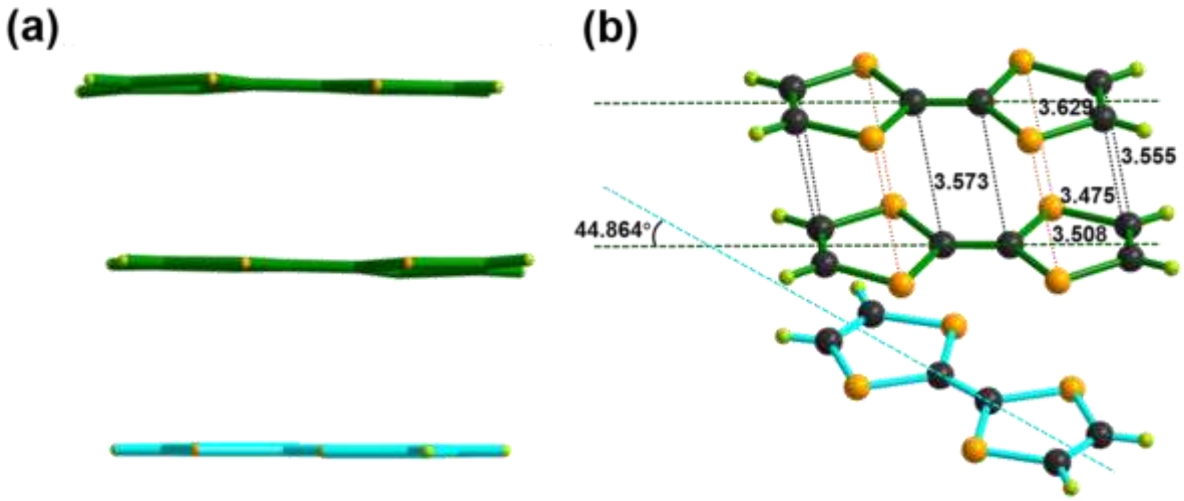

Figure S18. a) The geometry of the $TTF^{+\bullet}$ (green) and neutral TTF (blue) in the dicationic trimer. b) Detail structure of TTF dicationic trimer in TTF-$(TTF^{+\bullet})_2$-RC.

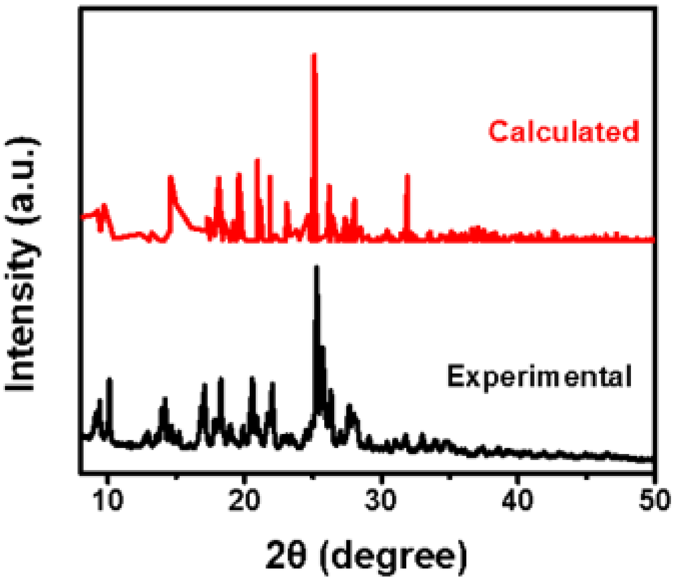

Figure S19. XRD spectra of experimental (black line) and calculated (red line) TTF-$(TTF^{+\bullet})_2$-RC.

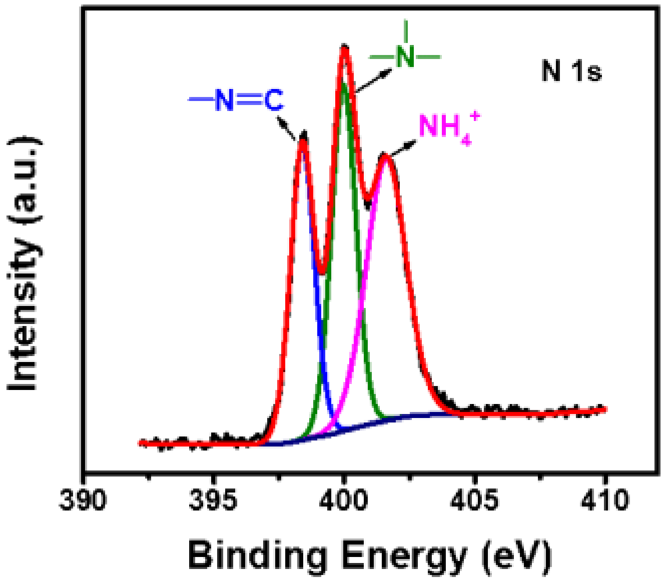

Figure S20. The XPS spectra of N 1s of ABTS powders.

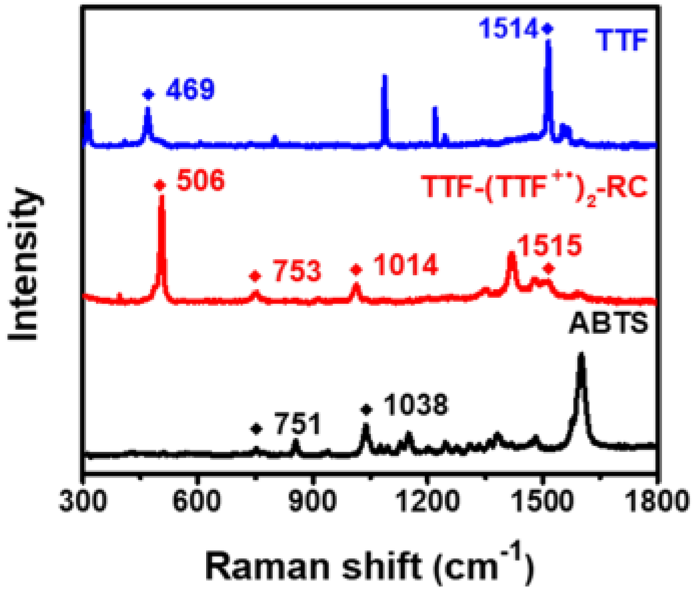

Figure S21. Raman spectra of TTF, ABTS, and TTF-($TTF^{+\bullet}$)$_2$-RC.

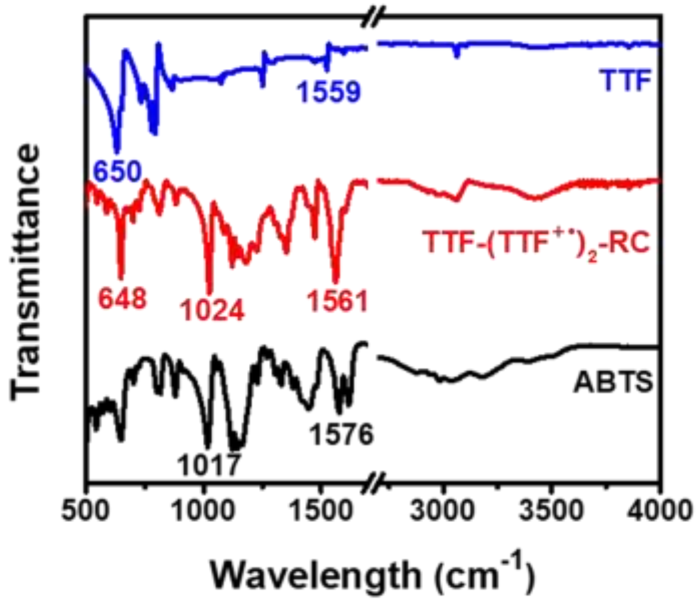

Figure S22. FTIR spectra of TTF, ABTS, and TTF-($TTF^{+\bullet}$)$_2$-RC.

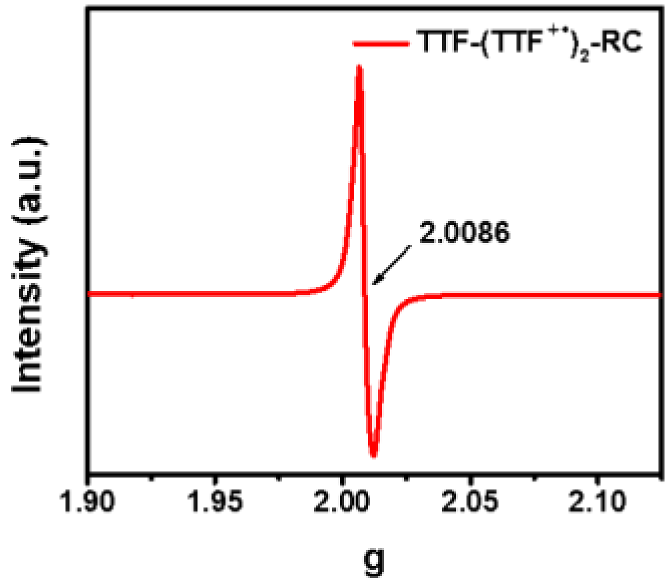

Figure S23. ESR spectra of crystalline TTF-($TTF^{+\bullet}$)$_2$-RC.

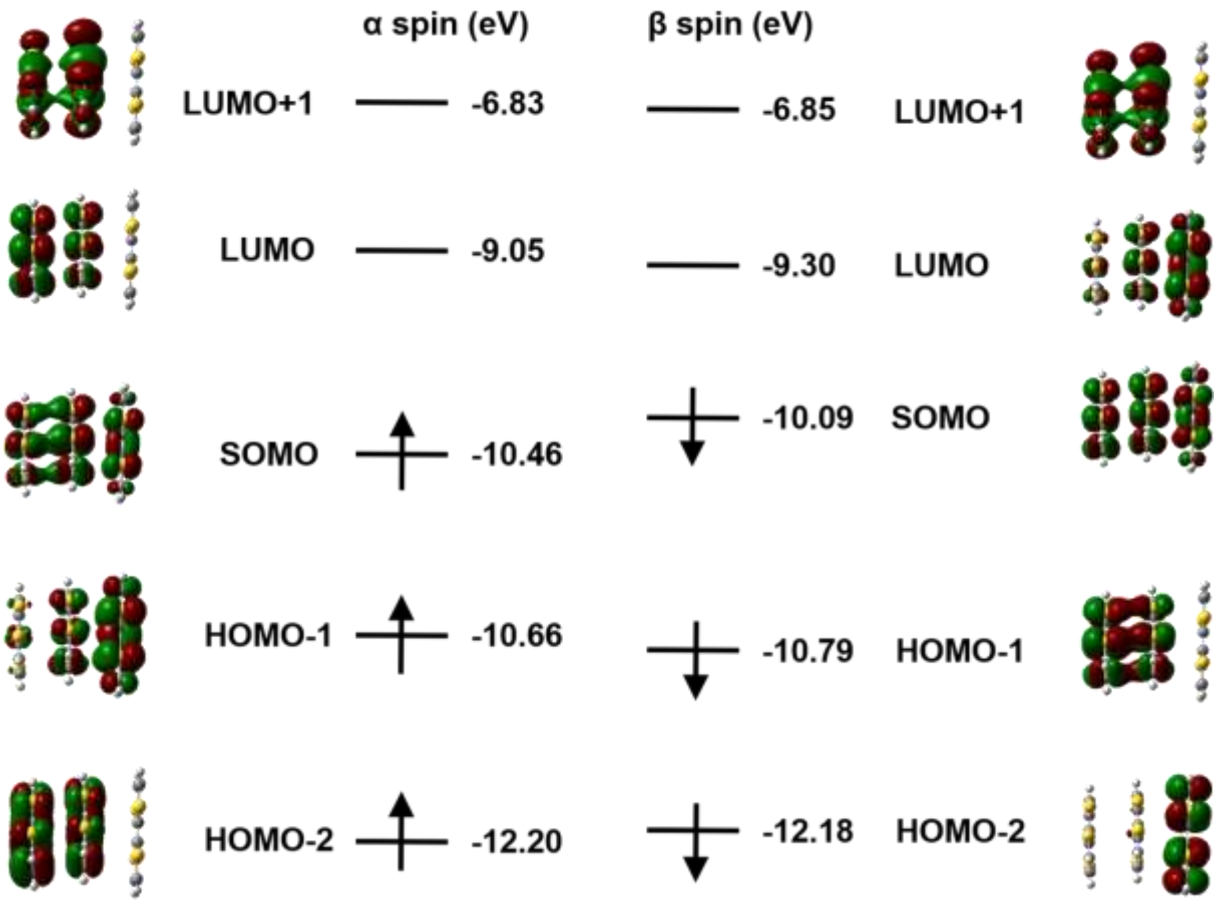

Figure S24. Frontier orbitals and corresponding energies of TTF dicationic trimer in TTF-$(TTF^{+\bullet})_2$-RC. Isocontour value is 0.04 a.u.

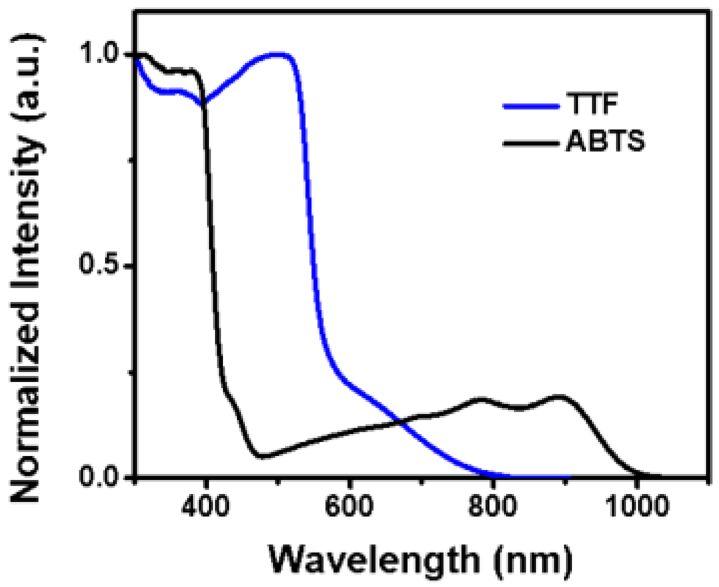

Figure S25. Normalized solid-state absorption spectra of TTF and ABTS.

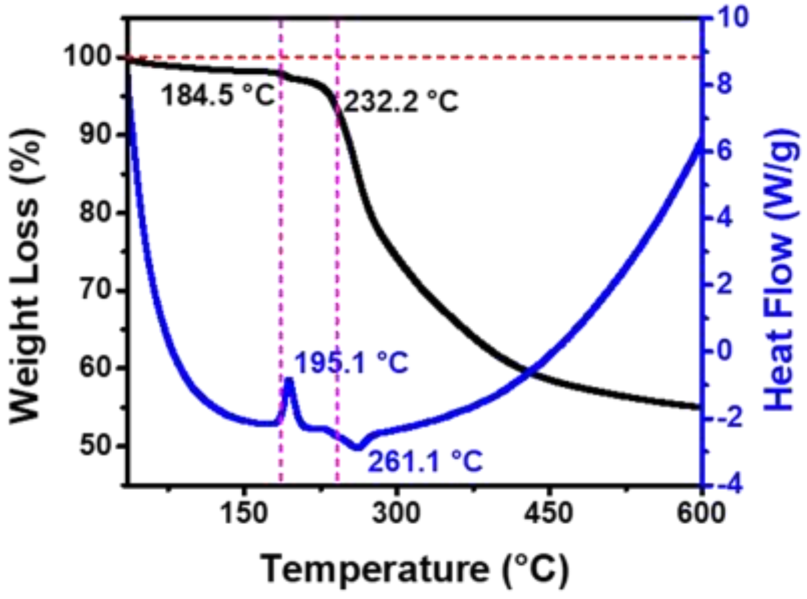

Figure S26. TG-DSC analysis of crystalline TTF-($TTF^{+•}$)$_2$-RC.

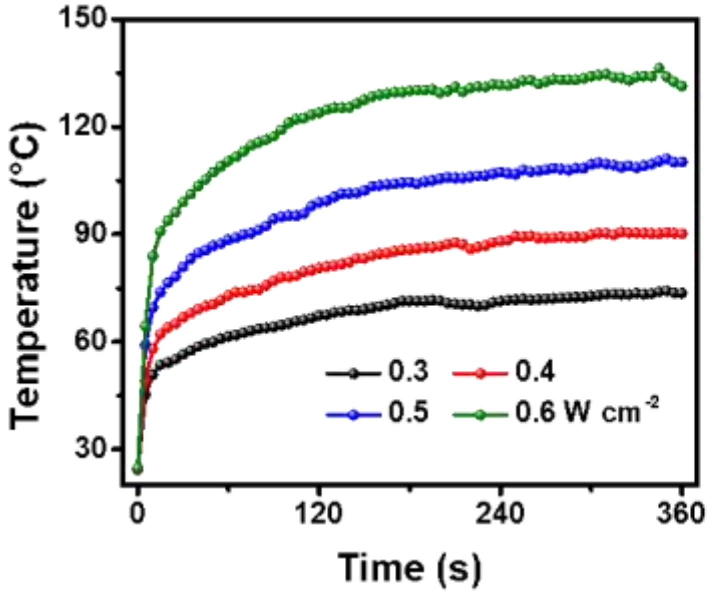

Figure S27. Photothermal heating curves of TTF-$(TTF^{+\bullet})_2$-RC powders under the irradiation of 1064 nm laser with different power densities.

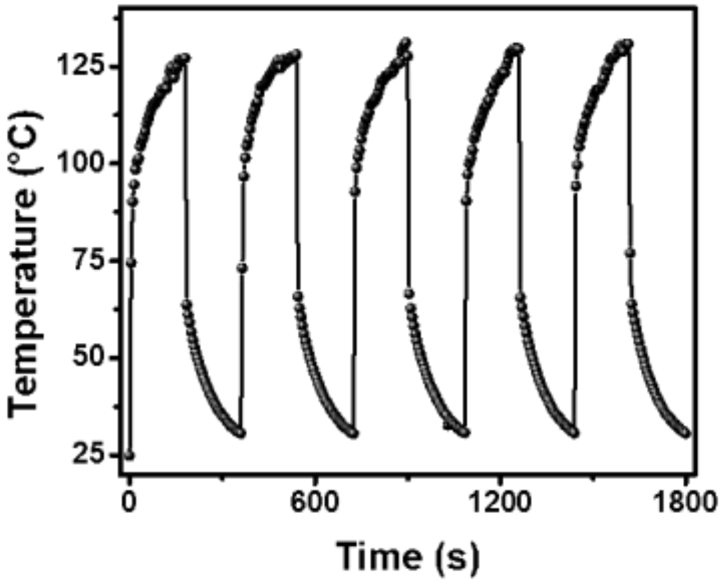

Figure S28. Photothermal cyclic curve of TTF-$(TTF^{+\bullet})_2$-RC powders under the irradiation of 1064 nm laser (0.6 W cm^{-2}).

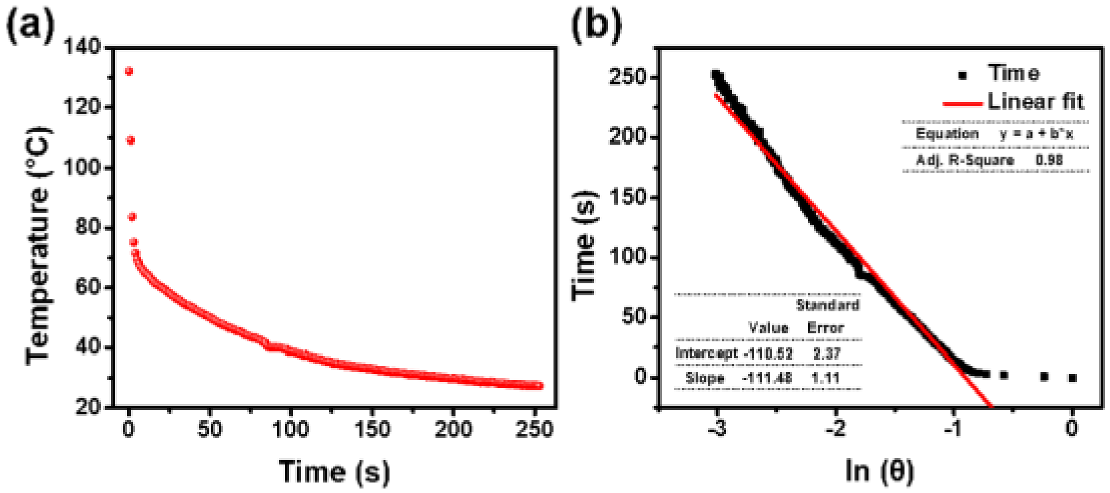

Figure S29. a) The cooling curve of TTF-($TTF^{+\bullet}$)$_2$-RC powders after the irradiation of 1064 nm laser (0.6 W cm^{-2}) and b) Its corresponding time-Inθ linear curve.

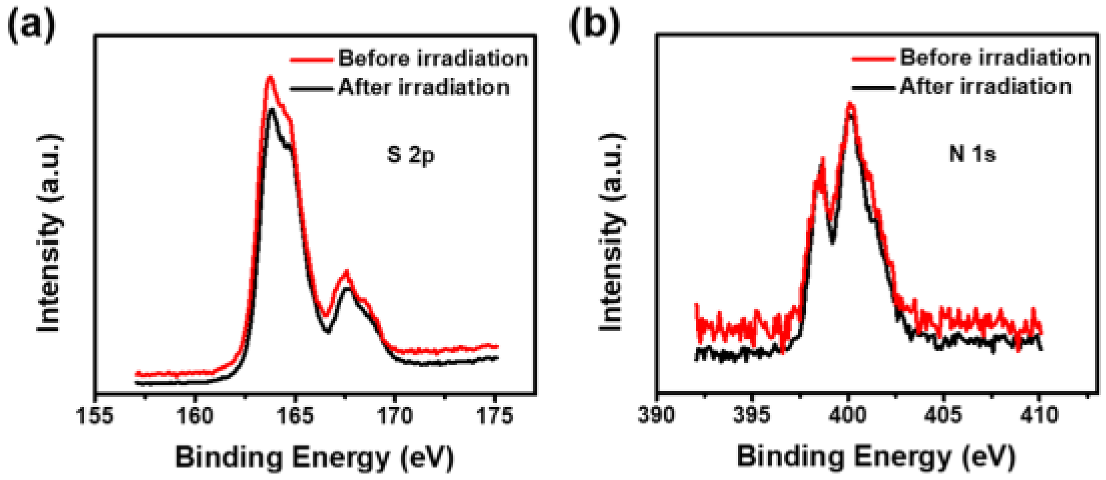

Figure S30. The XPS spectra of (a) S 2p and (b) N 1s of TTF-($TTF^{+\bullet}$)$_2$-RC before and after 1 h irradiation.

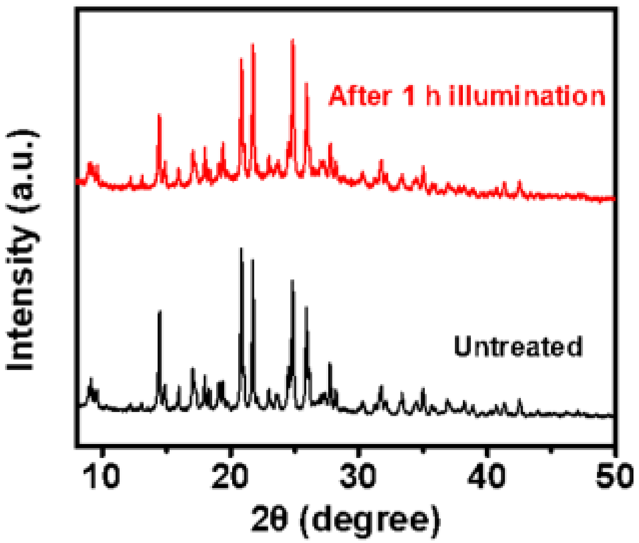

Figure S31. The P-XRD patterns of TTF-($TTF^{+\bullet}$)$_2$-RC before and after 1 h irradiation.

Table S1. Crystal data and structure refinement for TTF-(TTF$^{+\bullet}$)$_2$-RC.

Empirical formula	$\mathbf{C_{21}H_{18}N_2O_4S_{10}}$
Formula weight	682.97
Temperature/K	193.0
Crystal system	Triclinic
Space group	P-1
a/Å	10.6605(7)
b/Å	10.6656(7)
c/Å	13.3569(13)
α/°	106.614
β/°	101.906
γ/°	105.435
Volume/Å^3	1335.63(19)
Z	2
ρ_{calc}g/cm^3	1.698
F(000)	700
Crystal size/mm^3	0.05 × 0.01 × 0.01
Radiation	GaKα (λ = 1.34139)
2θ range for data collection/°	6.31 to 110.772
Index ranges	$-11 \leq h \leq 13$, $-12 \leq k \leq 13$, $-16 \leq l \leq 16$
Reflections collected	16599
Independent reflections	5063 [R_{int} = 0.0896, R_{sigma} = 0.0983]
Data/restraints/parameters	5063/0/338
Goodness-of-fit on F^2	1.019
Final R indexes [I ≥ 2σ (I)]	R_1 = 0.0771, wR_2 = 0.2061
Final R indexes [all data]	R_1 = 0.1290, wR_2 = 0.2416

Table S2. Bond length (Å) of TTF moieties in **TTF-(TTF$^{+\bullet}$)$_2$-RC**.

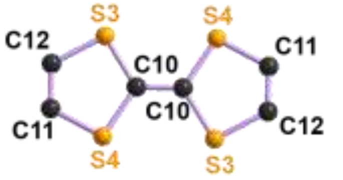

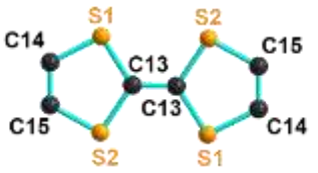

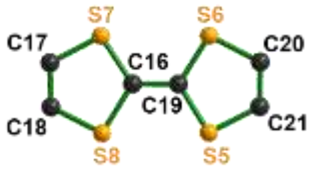

C(10)–C(10)	1.353(116)	C(13)–C(13)	1.334(93)	C(16)–C(19)	1.385(117)
S(3)–C(10)	1.764(66)	S(1)–C(13)	1.759(74)	S(5)–C(19)	1.714(64)
S(3)–C(12)	1.741(96)	S(1)–C(14)	1.740(91)	S(5)–C(21)	1.720(94)
S(4)–C(10)	1.758(95)	S(2)–C(13)	1.760(88)	S(6)–C(19)	1.714(82)
S(4)–C(11)	1.734(32)	S(2)–C(15)	1.730(81)	S(6)–C(20)	1.717(85)
Average S–C	1.749	Average S–C	1.747	S(7)–C(16)	1.718(68)
				S(7)–C(17)	1.732(112)
				S(8)–C(16)	1.744(91)
				S(8)–C(18)	1.718(99)
				Average S–C	1.722

Table S3. Average bond length and calculated charges (in atomic units) of TTF moieties in TTF-($TTF^{+\bullet}$)$_2$-RC. The results calculated by formula (2) were presented in parentheses.

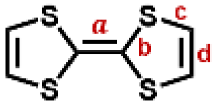

	Number	a, Å	b, Å	c, Å	d, Å	q, au
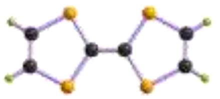	1	1.353	1.761	1.738	1.326	0.11 (0.12)
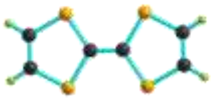	1	1.334	1.759	1.735	1.313	-0.09 (-0.08)
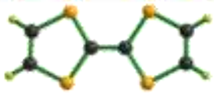	2	1.385	1.723	1.721	1.334	0.83 (0.87)

Table S4. Photothermal conversion efficiency for reported organic crystals materials.

PT materials	Laser wavelength (nm)	Power (W cm^{-2})	PCE (%)	Ref.
**TTF-($TTF^{+\bullet}$)$_2$-RC**	1064	0.6	62.9	This work
TMB-$ABTS^{+\bullet}$-H_2O cocrystals	1064	0.3	49.6	[10]
$2MPTC^{\bullet+}$-CB[8]	1064	1.5	54.6	[16a]
DBTTF-TCNB cocrystal	808	0.7	18.8	[29a]
TTF-Tri-PMDI cocrystal	808	0.7	15.0	[29b]
TMB-F_4TCNQ complex	1064	1.0	48.0	[29c]
TMB-TCNQ complex	1064	1.0	42.4	[29c]
Zr-$PDI^{\bullet-}$	1064	0.7	52.3	[29d]
Py-$BPy^{\bullet+}$-COF	808	1.0	63.8	[29e]
Py-$BPy^{\bullet+}$-COF	1064	1.0	55.2	[29e]
CR-TPE-T	808	0.8	72.7	[29f]

Reference

[1] Xu, J.; Chen, Q.; Li, S.; Shen, J.; Keoingthong, P.; Zhang, L.; Yin, Z.; Cai, X.; Chen, Z.; Tan, W. Charge-Transfer Cocrystal via a Persistent Radical Cation Acceptor for Efficient Solar-Thermal Conversion. *Angew. Chem. Int. Ed.* **2022**, *61*, e202202571.
[2] Brown, J. T.; Grounds, O.; Zeller, M.; Dilley, N. R.; Rosokha, S. V. Structures, Multicenter π-Bonding, and Spin Equilibria in the Mixed-Valence Trimers of Tetramethyltetrathiafulvalene Cation-Radicals. *Cryst. Growth Des.* **2021**, *21*, 7257–7268.
[3] Wang, Y.; Zhu, W.; Du, W.; Liu, X.; Zhang, X.; Dong, H.; Hu, W. Photothermal Conversion Cocrystals Strategy towards Materials for Near-Infrared Photothermal Conversion and Imaging. *Angew. Chem. Int. Ed.* **2018**, *57*, 3963–3967.
